# The Chemical Ecology Approach to Reveal Fungal Metabolites for Arthropod Pest Management

**DOI:** 10.3390/microorganisms9071379

**Published:** 2021-06-24

**Authors:** Alexander Berestetskiy, Qiongbo Hu

**Affiliations:** 1 All-Russian Institute of Plant Protection, Pushkin, 196608 Saint-Petersburg, Russia; 2 College of Plant Protection, South China Agricultural University, Guangzhou 510642, China; hqbscau@scau.edu.cn

**Keywords:** natural compounds, biorational insecticides, fungi, secondary metabolites, insecticidal proteins

## Abstract

Biorational insecticides (for instance, avermectins, spinosins, azadirachtin, and afidopyropen) of natural origin are increasingly being used in agriculture. The review considers the chemical ecology approach for the search for new compounds with insecticidal properties (entomotoxic, antifeedant, and hormonal) produced by fungi of various ecological groups (entomopathogens, soil saprotrophs, endophytes, phytopathogens, and mushrooms). The literature survey revealed that insecticidal metabolites of entomopathogenic fungi have not been sufficiently studied, and most of the well-characterized compounds show moderate insecticidal activity. The greatest number of substances with insecticidal properties was found to be produced by soil fungi, mainly from the genera *Aspergillus* and *Penicillium*. Metabolites with insecticidal and antifeedant properties were also found in endophytic and phytopathogenic fungi. It was noted that insect pests of stored products are mostly low sensitive to mycotoxins. Mushrooms were found to be promising producers of antifeedant compounds as well as insecticidal proteins. The expansion of the number of substances with insecticidal properties detected in prospective fungal species is possible by mining fungal genomes for secondary metabolite gene clusters and secreted proteins with their subsequent activation by various methods. The efficacy of these studies can be increased with high-throughput techniques of extraction of fungal metabolites and their analysis by various methods of chromatography and mass spectrometry.

## 1. Introduction

Phytophagous arthropods are a challenging problem in agriculture, horticulture, and forestry. Various cultural practices and the use of resistant plant varieties are among the traditional pest control methods but until recently the application of synthetic acaricides and insecticides had been the main solution. Due to side effects of the anti-insectan chemicals (for instance, on insect pollinators or parasitoids) and environmental pollution, the search for safer methods of controlling pest arthropods has been underway. The biological method of insect control based on the use of their parasites, predators, and pathogens (viral, bacterial, nematode, and fungal) is becoming increasingly accepted, especially in organic farming. However, efficacy of biocontrol agents is highly depended on number abiotic and biotic factors and often insufficient when compared to chemical control [1,2].

Another alternative to the synthetic chemicals is biorational insecticides based on natural compounds [3,4,5]. Table 1 summarizes the prospects of different groups of organic substances for the development of such the biorationals including entomotoxic compounds [6], insect growth regulators [7], and antifeedants [8]. Moreover, insecticidal proteins and their coding genes can be used to construct transgenic hypervirulent strains of entomopathogens [9] or pest-resistant crop cultivars [10].

Natural compounds have been active ingredients of a number of registered insecticides. Their sources are mainly soil bacteria and plants (Table 2), and the exponential increase in number of publications focused on the development of botanical insecticides should be noted [11]. Only recently, in 2018, the first insecticide, Inscalis^®^, based on a fungal metabolite was approved [12]. Meanwhile, among the known natural compounds having biological activity, almost half are substances of fungal origin, many of which have already found application in both medicine and agriculture [13]. Therefore, the fungal allelochemicals are still seemingly underestimated as potential biorational insecticides.

At the first stage of the screening programs, evaluation of extracts from natural materials (or from cultures of organisms) or pure compounds with various bioassays is performed [6]. One may utilize random material available or conduct targeted selection of organisms based on some principles, for example, using an ecological approach that demonstrated success in mining antibiotic producers [14,15].

The chemical ecology approach of the search for producers of anti-insectan substances may imply the presence of trophic and competitive relationships between arthropods and other organisms (fungi, in particular). Therefore, some ecological groups of fungi are supposed to produce some chemical antibiosis factors. From this point of view, there are some following assumptions. (1) Entomopathogenic fungi should produce virulence factors while soil micromycetes are expected to synthesize factors allowing colonization of soil or overwintering insects as well as defense substances against mycetophages. (2) Endophytic, phytopathogenic and coprophilic fungi are able of producing factors of antibiosis due to competition for the substrate with arthropods. (3) Macromycetes should produce toxic or deterrent metabolites for self-protection against mycetophagous organisms [16].

Recently, a significant number of theoretical papers have appeared on the direct and indirect (for example, through the host plant) chemical interaction of arthropods and fungi, for example, on the chemical mediators (mainly volatile compounds) of insect–fungal interactions [17], and on the metabolites of symbiotic, mutualistic, and entomopathogenic microorganisms associated with arthropods [18]. Both beneficial and phytopathogenic microbes associated with plants can alter the visual, olfactory, and gustatory cues of host plants, affecting the behavior and fecundity of phytophagous arthropods [19]; they can also stimulate plant defense responses directed against phytophages or the release of volatile metabolites that attract entomophages [20]. There are a few notable resumptive earlier publications on fungal insecticidal metabolites, such as [21], that focused on the toxins of soil fungi and [22] prospected allelochemicals for insect pest management.

Here, we summarize the scientific information on ecological groups of fungi and their bioactive metabolites (Table 1), which can affect the viability and fecundity of various pest arthropods (Table 3).

## 2. Low-Molecular-Weight Non-Volatile Compounds

### 2.1. Entomopathogens

Along with entomopathogenic viruses and bacteria, the fungi are a biotic factor limiting insect populations. Some entomopathogenic fungi (EPF) are considered or used in practice as bioinsecticides. Among them, special attention is paid to hypocrealean ascomycetes (order *Hypocreales*, class *Sordariomycetes*) and their anamorphs. At the same time, more than 80% of commercial mycoinsecticides are based on representatives of the genera *Beauveria* and *Metarhizium* [23]. It is considered that biologically active compounds (toxins and immunosuppressors) alongside lytic enzymes (proteases, lipases, and chitinases) are the virulence factors of EPF [24,25]. Therefore, when searching for insecticidal compounds, it is logical to pay attention to EPF first.

The structural diversity of biologically active substances detected in EPF is very large, and its detailed analysis is beyond of the tasks of this review. They are most well studied in fungi from the genera *Beauveria* (in particular, *Beauveria bassiana*, *B. brongniartii* and *B. felina*) and *Metarhizium* (*Metarhizium anisopliae*, *M. acridum*, *M. robertsii* and *M. brunneum*), as well as from the genera *Cordyceps*, *Paecylomyces*, and *Tolypocladium*.

Among small bioactive molecules produced by *Beauveria* spp., organic acids (oxalic acid), polyketides (oosporein), macrolactones (cephalosporolides), alkaloids (tennelin, bassianin, beauversetin, etc.), and cyclic depsipeptides (beauvericins, beauverolides, etc.) were well characterized [26,27,28,29]. Among them, the insecticidal activity of beauvericin, some beauverolides, and oosporein has been evaluated more or less intensively.

Numerous metabolites were detected in fungi of the genus *Metarhizium*, for instance, depsipeptides (destruxins), alkaloids (fungerins, cytochalasins, swainsonine), terpenoids (viridoxins, ovalicin), siderophores (metachelins), polyketides (aurovertins, kojic acid) [30,31,32]. Metabolomic studies have also revealed known alkaloids (hirsutellones A–C and some ergoalkaloids), macrolactones (torrubiellutins A–C), naphthoquinones (naphthgeranins B–D), trichothecans (spirotenuipesines A, B) [33,34]. Insecticidal properties are known for a few of the following compounds: destruxin A and E, viridoxin A and some others.

Among the secondary metabolites of fungi of the genus *Cordyceps sensu lato*, cordycepin, phomalactone and beauvericin produced by *Cordyceps militaris*, *C. cicadae* and *Ophiocordyceps communis*, respectively, demonstrated anti-insectan properties [35,36].

*Beauvericin.* The cyclooligomeric depsipeptide beauvericin is the trimer of the dipeptidol monomer of D-hydroxyisovaleric acid. It has been detected in some EPF species from the genera *Beauveria*, *Cordyceps*, and *Isaria*, as well as in some species of phytopathogenic fungi of the genus *Fusarium* [27,37,38]. Beauvericin is found mainly in the mycelium of *B. bassiana*, as well as in conidia formed on the infected insect body, but not in blastospores [39].

The blowfly *Calliphora erythrocephala* (up to 15% mortality at a concentration of 5 µg/individual) and larvae of the malaria mosquito *Aedes aegypti* (the mortality level was 86% at a concentration of 20 µg/mL) were sensitive to beauvericin [40]. Being of low toxicity for the wheat aphid *Schizaphis graminum* at a concentration of 500 µg/mL, beauvericin significantly reduced the pest fertility. Histological studies showed the toxin to inhibit aphid bacteriocytes by binding to DNA of endosymbionts [41]. There are more than 20 natural compounds structurally similar to beauvericin with lower or unknown insecticidal activity [42,43].

Beauvericin showed strong acaricidal properties (lethal dose 50%, LD_50_ 0.65 µg/mL) against the two-spotted spider mite *Tetranychus urticae* comparable to the efficacy of commercial acaricides (bifenazate and cyflumetofen). It was non-phytotoxic for protected strawberries at a rate of 160 g/ha. However, after 40 generations, the sensitivity of the mites to the toxin dramatically decreased [44].

Beauvericin has significantly higher cytotoxic activity against the fall armyworm *Spodoptera frugiperda* Sf9 (LD_50_ 2.8 µg /mL) and Sf21 (LD_50_ 6.9 µg /mL) cell lines than other *Beauveria* spp. toxins as bassianin, oosporein, and tennelin [45]. It also exceeds cytotoxicity of a number of mycotoxins as gliotoxin, nivalenol, enniatin, zearalenone, deoxynivalenol when tested on the same Sf cell lines [46].

This toxin has the ionophore properties and a wide range of cytotoxic and antibacterial activity. It increases the concentration of calcium ions in the cytoplasm and leads to the decrease in ATP content and to the calcium-dependent pathway of cell apoptosis. Beauvericin is able to enhance the action of antimycotics and cytostatics against multiple-resistant forms of *Candida albicans* and various tumor cell lines, respectively [47]. Thus, with its own low insecticidal activity, it may sensitize insect cells or symbiotic microorganisms to bioactive metabolites of EPF.

Beauvericin encapsulated in chitosan nanoparticles showed higher activity against taro caterpillar *Spodoptera litura* compared to “free” beauvericin [48]. However, it is important to note that beauvericin is considered as a mycotoxin of some *Fusarium* spp. and, therefore, a pollutant of food and feed [49,50]. In addition, beauvericin has phytotoxic properties when accumulated in plant cells [51]. Apparently, beauvericin toxicity will significantly complicate its implementation in plant protection as natural insecticide. Moreover, the yield of beauvericin under optimized culture conditions was approximately 400 mg/L of medium, which seems insufficient for the commercialization of the product [47].

*Beauverolides* is a family of cyclic tetradepsipeptides characterized by the presence of a 3-hydroxy-4-methyl-carboxylic acid residue. The compounds were isolated from *B. bassiana*, *B. tenella* (= *B. brongneartii*), *Cordyceps militaris*, and *P. fumosoroseus* (=*Isaria fumosorosea*). Beauverolide A is one of the virulence factors of these EPF [52]. Beauverolide A at 10 µg per larva showed weak insecticidal activity against the taro caterpillar *S. litura* (20% mortality) and the Chinese bruchid *Callosobruchus chinensis* (death of 40% of females) with the absence of antimicrobial activity [53]. When administered intragemocellularly (10 µg per larva), beauverolide L, along with cyclosporine A, did not cause the death of the greater wax moth *Galleria mellonella*, but changed their immune status by inhibiting the phagocytic activity of plasmatocytes, at the same time stimulating the humoral immune response [54].

*Oosporein* is a derivative of 1,4-dibenzoquinone found in the culture of various micromycetes, including some species of the genus *Beauveria*. This toxin is one of the main metabolites of *B. brongniartii* and was detected in diseased insects [55,56]. Oosporein-producing strains of *B. bassiana* are highly pathogenic [57]. Crude oosporein (0.3 mg/mL) was slightly toxic to the whitefly *Trialeurodes vaporariorum*, but when mixed with *B. bassiana* spores, the effect was synergistic [58]. When added to the artificial diet, oosporein showed neither repellent nor insecticidal properties against the larvae of *G. mellonella* and the white grub cockchafer *Melolontha melolontha* [59].

Oosporein demonstrates cytotoxic and antioxidant properties, as well as a wide range of antimicrobial activity. During the development of infection caused by *B. bassiana*, oosporein suppresses the immunity of insects, inhibiting the prophenol oxidase cascade and the expression of genes responsible for the synthesis of antifungal peptides. There was a significant decrease in the number of bacteria in the *G. mellonella* larvae that dead from mycosis [60,61,62,63].

*Destruxins* are a group of cyclic hexadepsipeptides that have been well studied. Among several dozen structurally related compounds, destruxins A and E are the most toxic to insects. Extracts from the *Metharhizium brunneum* culture containing destruxin A and destruxin A2 effectively controlled the Mediterranean fruit fly *Ceratitis capitata* [64]. Various formulations of destruxin A were effective both in the laboratory and in the field against the peach aphid *Myzus persicae* [65] and the sugar beet fly *Pegomya mixta* [66].

Destruxin A (DA) is assumed to suppresses the innate immune response in insects infected with *Metharhizium* spp. [67,68]. DA was cytotoxic to granulohemocytes and plasmatocytes of the silkworm *Bombix mori* in vitro with the LC_50_ values of 69 and 84 µg/mL, respectively. In vivo the hemocytes were more susceptible to DA at the extremely low dose of 0.25 µg/mL hemolymph [69]. Several silkworm proteins were found to bind DA, e.g., arginine tRNA synthetase and the stress support protein Lamin-C [70].

Biotechnology of production of destruxins has been improved. Under the optimized conditions for selected strains of *Metharhizium* spp., the yield of destruxins A and B reaches 200–500 mg/L [71,72,73]. However, due to the relatively weak insecticidal activity and relatively low yield in the fungal cultures, the direct use of destruxins is unlikely.

*Viridoxins* A and B from the group of diterpene pyrones showed high insecticidal activity by the leaf disk assay against larvae of *Leptinotarsa decemlineata* (Colorado potato beetle) with LD_50_ of 40 and 50 µg/mL, respectively. Structurally, viridoxins are similar to phytotoxins of the colletotrichine group, which, surprisingly, do not possess anti-insectan properties. The yield of viridoxins was low (10 mg/L) and no further publications were found on them [74].

*Cordycepin* is one of the main metabolites of *C. militaris* [75] that shows cytotoxic activity via termination of the synthesis of nucleic acids [76]. Data on the insecticidal properties of this toxin are limited. At intragemocellular injection, LD_50_ of cordycepin for *G. mellonella* larvae was 30 µg/g [77]. Diamondback moth *Plutella xylostella* larvae died after 5 days of their incubation on leaf disks treated with the toxin at a concentration of 0.3–0.5 mg/mL. At the same time, it was non-toxic when applied topically [78]. Cordycepin was detected in artificially infected *G. mellonella* larvae to be an inhibitor of their immune response (e.g., the expression of protective genes) contributing to the disease development [79,80].

Phytotoxic properties cordycepin were reported to propose it as a natural herbicide [81]. Although cordycepin can be produced on an industrial scale for medical purposes [82], its use as an insecticide is unlikely to be appropriate.

*Phomalactone*, which was also isolated from the entomopathogenic fungus *Hirsutella thompsonii* var. *synnematosa*, was toxic to the imago of the fly, apple maggot *Rhagoletis pomonella* when added to a liquid feed at a concentration of 2 mg/mL [83]. Its insecticidal properties against the malaria mosquitoes *Ae. aegypti* were shown with LD_50_ 0.64 µg per individual [84]. The insecticidal properties of phomalactone and its producer (*Paecilomyces cateniannulatus* YMF 1.01773) were protected by the PRC Patent N CN102532081B. However, the yield of this metabolite is low (up to 100 mg/L) in *Ophiocordyceps communis* culture liquid [35]. In addition, phomalactone displays other types of biological activity, for example, high fungicidal activity (MIC 2.5 µg/mL) against the causative agent of the potato late blight, *Phytophthora infestans* [85].

*Cyclosporine A*, an antifungal cyclic polypeptide, which was originally found in *Tolypocladium inflatum*, has been introduced into medical practice as an immunosuppressant [86,87]. The compound was found to be highly toxic for *Ae. aegypti* mosquitoes [88]. This toxin has been shown to reduce the activity of lysozyme and antimicrobial peptides in *G. mellonella* larvae treated with cyclosporin A, increasing their susceptibility to microbial infection [89]. Despite cyclosporine A can be produced in sufficient quantities its development as an insecticide is unlikely because of the preferable use in the medicine.

Analysis of the spectrum of biological activity of the metabolites identified in the well-known EPF revealed the interesting fact that most of them does not possess specific insecticidal activity. Minor EPF metabolites have generally not been evaluated as insecticidal compounds at all. Destruxins, beauvericin, cordycepin and other substances have showed immunosuppressive properties and, apparently, serve as important factors in the development of insect mycoses. The latest review work on the metabolites of hypocrealean ascomycetes seems to have confirmed this supposition [90]. It is important to note that insect immunomodulators are also produced by entomopathogenic bacteria [91] and nematodes [92].

Many EPF synthesize metabolites are just slightly toxic to arthropods with pronounced antimicrobial properties, such as oosporein. This is interesting because some antibiotics, such as doxycycline, may have a direct insecticidal effect [93]. In addition to the representatives of the mentioned above EPF genera, antibacterial and antifungal metabolites were found in *Lecanicillium* sp. [94], *Akanthomyces gracilis* [95], *Gibellula* sp. [96], and many others. Their function is obviously related to the survival of their producers in the soil and to inhibit the gut microflora of insects, which may have antagonistic properties with respect to EPF [97,98].

Studies on the search for inhibitors and antagonists of juvenile hormones produced by EPF are sporadic. Screening of more than a hundred extracts from cultures of various types of EPF did not lead to the detection of agonists of juvenile hormones, while approximately 10% of the tested extracts had antagonistic activity against these hormones. An extract of the *Lecanicillium attenuatum* strain JEF-145 displayed the highest level of antihormonal and insecticidal activity against *Aedes albopictus* and *P. xylostella* [99].

### 2.2. Soil Fungi

Micromycetes of the genera *Aspergillus*, *Fusarium*, *Penicillium*, *Trichoderma*, and others are most often isolated from soil samples. Sometimes the fungi have been isolated from died insects and can even be causal agents of their mycoses. For instance, the virulence of *A. flavus* against *G. mellonella* larvae [100], *F. larvarum*, *F. proliferatum* and *T. harzianum* against aphid *S. graminum* [101], *F. solani* and *T. harzianum* for the American cockroach *Periplaneta americana* [102], *F. subglutinans*—the Western flower thrips *Frankliniella occidentalis* [103] *Aspergillus* and *Fusarium* spp.—against the silverleaf whitefly *Bremisia tabaci* [104] was proved. Since soil fungi have long been known as producers of biologically active compounds, one can assume their toxigenic effect on arthropods. On the other hand, their insecticidal metabolites can be considered as protection factors against mycetophages, since they mainly accumulate in sclerotia (resting mycelial modifications), ascostroma, or in vegetative mycelium post its mechanical damage [105,106]. Fumagillin, gliotoxin, and ergoalkaloids have been shown to play an important role in the infection of *G. mellonella* larvae with *A. fumigatus* [107,108,109].

Gloer (1995) [110] summarized data from early studies on the screening and isolation of insecticidal metabolites from sclerotia of soil micromycetes. The approach was justified by observations on behavior of mycetophagous insects (for example, the fruit fly *Drosophila melanogaster*), which avoided sclerotia on *Aspergillus* culture plates, preferring to feed on mycelium [111]. Further, significant differences were noted in the qualitative composition of extracts from sclerotia and mycelium of some aspergilli. From the resting structures of *Aspergillus* spp., about 100 new compounds were isolated, most of which demonstrated anti-insectan activity [110]. A number of them had been patented as potential insecticidal molecules: aspernomine [112], sulpinine, secopenitrem B, aflatrem B [113], leporin A [114], cycloechinulin [115].

Recent studies have confirmed the induction of sclerotia formation in aspergilli culture is related to biosynthesis of further novel metabolites. Growing *A. sclerotiicarbonarius* IBT 28362 on a nutrient medium that stimulates the sclerotia formation, several substances with insecticidal activity against *D. melanogaster* larvae (LD_50_ 1.8 µM) were detected [116,117]. Asperparalins (tetramic acid derivatives) found in *A. japonicus* JV-23 causes paralysis in the silkworm *B. mori* [118]. In particular, asperparalin A at a concentration of 100 nM selectively blocks the nicotine-sensitive choline receptor of this insect [119].

Study of the chemistry of *Eupenicillium* ascostroma also gave interesting results. They contain mainly metabolites characteristic of *Aspergillus* sclerotia. New anti-insectan compounds were also identified, for example, shearinin B in *E. shearii*, which led to a significant growth inhibition of the corn earworm *Heliothis zea* larvae at a concentration of 100 µg/g in pinto bean diet bioassay and 84% mortality of the of *S. frugiperda* caterpillars in leaf disc bioassay at a concentration of 50 µg/disc [109,120]. Other insecticidal metabolites have also been isolated in penicilli, for example, penitrems A‒B and their natural analogues. In particular, penitrem A was patented as a broad-spectrum insecticidal molecule [121].

The most interesting finding among the metabolites of aspergilli and penicilli was the discovery of pyripyropene A from the group of meroterpenoids in *A. flavus* [122]. Initially selected as an inhibitor of the acyl-CoA-cholesterol-acyltransferase, it showed strong insecticidal properties. Pyripyropenes have been detected also in *P. coprobium*, *P. griseofulvum* and some other micromycetes. The semisynthetic derivative of pyripyropene A, afidopyropene recently was registered as an insecticide Inscalis^®^ by BASF. Afidopyropene blocks activity of the TRPV-ion channels of the chordotonal organs of insects, disrupting their coordination and nutrition [123].

Substances that disrupt the insect molting were isolated from the cultures of some soil fungi. Castillo and co-authors (1999) [124] tested extracts from cultures of 21 isolates belonging to 11 species of *Penicillium* spp. for insecticidal and anti-juvenile activity. Significant inhibition of juvenile hormone (70‒75% of prematured imago) was demonstrated by dichloromethane extracts from the culture fluid of two *P. brevicompactum* strains at a concentration of 10 µg/cm^2^. The addition of a juvenile hormone analog to the nutrient substrate leveled the effect of fungal metabolites on insects. Two active compounds from the ketoamide group were isolated from the extract of the culture fluid of these strains. These ketoamides were synthesized and showed the same properties as the natural substances [124,125]. Moreover, some of their synthetic analogues showed higher insecticidal activity than their natural prototypes [126]. An analog of juvenile hormone III was isolated from *A. nidulans*. Feeding *D. melanogaster* larvae on the fungal culture stimulated the formation of this compound and slowed down the development of insects compared to the control [127]. The fungus may be supposed to hold the potential host at feeding and susceptible stage to get more resources from it for better survival or sporulation.

Fungi of the genus *Trichoderma* are common antagonists and hyperparasites of fungi. Numerous metabolites with a wide range of biological activity were found in representatives of this genus [128,129]. The aphid *S. graminum* recognizes the metabolites of *Trichoderma* spp. [130]. Among of them (citrantifidiene, citrantifidiol, dihydrotrichodimerol and bislongiquinolide) at a concentration of 0.5–1 mg/mL showed deterrent effect against this pest [131,132]. Long-chain alcohols (in particular, 1-hexadecanol) found in the mycelium of *T. citrinoviride* have been patented as potential agents for controlling the bird cherry-oat aphid *Rhopalosiphum padi* [133]. Interestingly, substances from this group are part of the sexual pheromones of various insects [134].

Evaluation of ethyl acetate extracts from cultures of 142 isolates belonging to 50 species of the genus *Chaetomium*, conducted on larvae of *S. frugiperda* and imago of the dried fruit beetle *Carpophilus hemipterus*, demonstrated relatively high insecticidal activity of most of them. However, a few of novel metabolites were revealed in the extracts while known mycotoxins and immunosuppressants (for example, sterigmatocystin, chaetoglobosins and cyclosporin A) were their major components. The maximum insecticidal activity was demonstrated by sterigmatocystin against European maize borer *Ostrinia nubilalis* at a concentration of 60 µg/g [135].

Fungi of the genus *Fusarium* have been isolated from dead insects, and some of them are able to cause mycoses. The entomotoxic properties of fusariotoxins were studied extensively in the 1970s and 1980s [136]. For instance, among the dihydroisocoumarins isolated from *F. larvarum* culture, monocerin, mellein, and fusarentin 6-methyl ether showed relatively high activity against *C. erythrocephala* with LD_50_ of about 50 µg/mL [137]. Mycotoxin fumonisin FB from *F. verticillioides* did not affect viability but caused repellent effect on the maize weevil *Sitophilus* [138]. Among the sterols isolated from *Fusarium* sp. culture, sterol sulfate inhibited the growth of *H. zea* and *S. frugiperda* larvae at concentrations of 2.5 mg/g and 4 mg/g of feed, respectively [139].

Many soil micromycetes (already mentioned genera *Aspergillus*, *Fusarium*, *Penicillium*) produce mycotoxins, substances that are toxic to animals. Colonizing plant residues or food products (grain, vegetables, fruits, etc.) during storage, the fungi form toxins, apparently, to protect the substrate from insect pests. These natural substances had also been evaluated for insecticidal activity [21].

Aflatoxin B1 at a concentration of 10‒100 mg/kg of the nutrient medium showed significantly higher larvicidal activity against *D. melanogaster* than rubratoxin B (RTB) and diacetoxiscirpenol (DSL), while patulin (PT) was just slightly toxic. At a concentration of 1 µg/cm^2^, RTB and PT showed strong contact insecticidal activity against the fruit fly, while aflatoxin B1 and DSL were of low toxicity [140]. A nitrogen-containing coumarin derivative, mycotoxin ochratoxin A (OTA), was detected in sclerotia of *A. carbonarius*, grown on corn grain, in a concentration of about 50 mg/g of dry sclerotia. When the toxin was added to the artificial feed at a dose lower than in the sclerotia, it led to a 75% reduction in feeding rate of *C. hemipterus* imago and to 50% mortality of larvae of the corn earworm *Helicoverpa zea* with a considerable reduction in the weight of the survived individuals [111]. 

The number of mycotoxins, namely, OTA, RTB, PT, citrinin (CT), penicillic acid (PA), and oxalic acid, when added to wheat flour, had different effects on the development of various insect pests of stored food products: the confused flour beetle *Tribolium confusum*, the tobacco beetle *Lasioderma serricorne*, and the black carpet beetle *Attagenus megatoma*. OTA and CT inhibited the growth of *A. megatoma* larvae at concentrations of 10 and 1000 mg/kg, respectively, while they were not affected by RTB. PT, CT, and RTB only inhibited the development of *T. confusum* and *L. serricorne* at a concentration of 1000 µg/g. The reproductive function of *T. confusum* was reduced when its feed contained CT, PT, and OTA, while only CT affected the fecundity of *L. serricorne* [141]. Dowd (1989) evaluated the oral toxicity of CT, OTA, and PA against the larvae of *H. zea* and *S. frugiperda*, at concentrations that are commonly found in nature. OTA and CT were the most toxic and caused abnormalities in the development of the malpighian tubules in the caterpillars. The combination of OTA and PA acted synergistically against *H. zea*, whereas the combination of OTA and CT showed synergistic insecticidal effect on *S. frugiperda* [142]. Worker bees of *Apis mellifera* were highly sensitive to aflatoxin B1 and OTA at concentrations of 10 µg/g and 5 µg/g, respectively [143]. Among the three mycotoxins (T-2, deoxyvalenol, and zearalenone), only the last had a significant effect on the fertility of aphid *S. graminum* at a concentration of about 10 µg/cm^2^ [144]. 

Recently, many studies on the tolerance of phytophages to mycotoxins have appeared. For example, the colonization of wheat by aphids was found to stimulate the garin infestation with fusaria and subsequent accumulation of T-2 and HT-2 toxins [145]. Damage of nuts by the navel orangeworm, *Amyelois transitella*, is often accompanied by their contamination by aspergilli molds and, consequently, with their mycotoxins. This pest of peanuts, being in contact with mycotoxins was significantly (LD_50_ is about 100 times higher) more tolerant to aflatoxin B1 than *H. zea* [146].

Some pests of stored food products can feed infected substrates without significant damage for themselves. For instance, the yellow mealworm *Tenebrio molitor* use grain contaminated with beauvericin, enniatin, and fumonisins [147]. *T. molitor* larvae did not accumulate deoxyvalenol (DON) during feeding on the grain containing this mycotoxin at a level about 5 µg/g [148]. The wheat aphid *Sitobion avenae* is capable of transforming DON to the less toxic DON-3-glucoside [149]. Food protein producers, in particular, the Buffalo beetle *Alphitobius diaperinus* and the black soldier fly *Hermetia illucens*, are able to excrete or metabolize certain alflatoxin B1, DON, OTA, and zearalenone [150].

Due to the high general toxicity, the use of mycotoxins against pest arthropods is unlikely. Moreover, in the process of coevolution, some insects became insensitive to common fungal toxins. The biochemical mechanisms of deactivation of mycotoxins by insects can be used for the development of new food decontamination methods [151].

### 2.3. Endophytes

Endophytic fungi have attracted strong interest as producers of biologically active substances. These microorganisms, including some entomopathogenic species, grow asymptomatically inside plants [152,153,154]. Endophytes are assumed to produce various metabolites assisting the host plant to restrain the development of phytopathogens and phytophages [155,156]. The literature survey revealed the growth and productivity of polyphagous and sucking insects to be significantly reduced on plants colonized by endophytes whereas the specialized phytophages seems overcome the chemical defense barriers being less sensitive to plant/endophyte bioactive metabolites [157].

Fungal endosymbionts of grasses belonging to the genera *Neotyphodium* and *Epichloë* produce numerous alkaloids, some of which are toxic to grazing cattle and/or insects. For instance, ergoalkaloids (ergoline derivatives) and lolitrems (indolditerpenes) have a neurotoxic effect on animals; lolines (1-aminopyrrolizidines) have broad-spectrum nicotine-like insecticidal properties while peramine (pyrrolopyrazines) demosntrates antifeedant properties [158,159,160]. The content of lolins (in particular, N-formyl-lolin and N-acetyl-lolin) in the tissues of fescue species varied in concentrations from 70 to 500 µg/g and correlated with plant resistance against two aphid species *R. padi* and *S. graminum* [161]. Similarly, at a high content of these alkaloids in the stems of ryegrass colonized by *Neotyphodium uncinatum*, the growth and development of larvae of the Argentine stem weevil *Listronotus bonariensis* was considerably slowed [162]. The content of peramine increases sharply in mechanically damaged leaves of ryegrass (*Lolium perenne*), for example, in drops of liquid released at the cut sites of stems and leaves, while the toxin was not detected in the exudate of intact leaves [163]. Production of lolins in the culture of *N. uncinatum* is a relatively high on a synthetic medium with asparagine as a nitrogen source with a yield about 700 mg/L [164].

The spruce budworm *Choristoneura fumiferana*, being one of the most dangerous pests of conifers in North America [165], was selected as a target for screening of anti-insectan metabolites produced by endophytic fungi isolated from needles of some spruce species (*Picea mariana*, *P. rubens*, *P. glauca*, etc.), balsam fir (*Abies balsamea*) and American larch (*Larix laricina*). Interestingly, approximately 20% of the fungal extracts with insecticidal activity produced anthraquinone rugulosin. When added to an artificial diet at the level of 10–25 µM, rugulosin led to a significant slowdown in the development of *C. fumiferana* caterpillars. The lowest concentration of this toxin, which affects insects, was about 0.5 µg/g of conifer needles. Treatment of small trees with the fungus *Phialocephala scopiformis*, which produces rugulosin as a main metabolite, reduced the development of the pest and increased the tolerance of conifers to *C. fumiferana*. However, the use of this substance itself is very doubtful since it has a strong toxicity to mammals [166,167,168]. In addition to rugulosin, some other anti-insectan metabolites of endophytic fungi can be noted. Several insecticidal derivatives of isocoumarin, structurally similar to ramulosin and mellein, were isolated from *Conoplea elegantula*, an endophyte of black spruce (*P. muriana*) [169]. Heptedelic acid from the culture of *Phyllosticta* sp. was active against *C. fumiferana* caterpillars at a concentration of 0.2 µM [170]. Phomopsolides A and B isolated from *Diaporthe maritima* (an endophyte of some spruce species) and *Phomopsis oblonga* have a deterrent effect on the larvae of the large elm bark beetle *Scolytus scolytus* [171,172].

Less is known about the insecticidal properties of endophytes of dicot plants. Ethyl acetate extract from the culture of *Cladosporium uredinicola*, isolated from the vine, heart-leaved moonseed (*Tinospora cordifolia*), significantly slowed down the development of *S. litura* larvae when added to artificial feed at a concentration of about 2 µl/g [173]. Nodulisporic acid A, an indolterpene alkaloid, isolated from the culture of endophytic *Nodulispora* sp., was toxic to *Ae. aegypti* mosquito larvae with an LD_50_ of 0.5 µg/g and larvae of the common green bottle fly *Lucilia sericata* with an LD_50_ of 0.3 µg/g. The efficacy of the ivermectin used as a positive control was significantly higher: LD_50_ 0.02 and 0.045 µg/g, respectively [174]. Extracts of *Talaromyces pinophilus* isolated from the strawberry tree (*Arbutus unedo*), had insecticidal activity against the pea aphid *Acyrthosiphon pisum*. The metabolomic analysis of the extracts allowed to identify the siderophore ferrirubin, the platelet aggregation inhibitor herculin B, and the antibiotic 3-O-methylfunicon. The latter demonstrated a certain toxic effect (aphid death at the level of 25% compared to the control) 3 days after spraying at a concentration of 50 µg/mL [175]. Two metabolites of *Diaporthe miriciae* (*Cyperus iria* endophyte), phyllostin and phyllostin acetate, showed significant (50% of the tested individuals refused to eat on the treated cabbage leaf discs at a concentration of 9 and 4.7 µg/cm^2^, respectively) contact insecticidal activity (LD_50_ 4.4 and 6.5 µg/individual) against *P. xylostella* larvae, and also reduced the fecundity of this pest [176].

### 2.4. Phytopathogens

There are few reports of anti-insectan metabolites of phytopathogenic fungi. These substances may be necessary for them to compete with phytophagous arthropods for a plant substrate. 

Extracts from the cultures of two wheat pathogens, *Parastagonospora nodorum* and *Bipolaris sorokiniana*, demonstrated aphidocidal activity, presumably due production of mycophenolic acid and sterigmatocystin, respectively [177]. Monocerin, a common metabolite of a number of phytopathogenic fungi from the genus *Exserohilum*, has insecticidal activity along with antifungal and phytotoxic properties [137,178]. The toxin was found in another phytopathogenic fungus, *Macrophomina phaseolina*, which also produces other entomotoxic compounds such as mellein, cordycepin, and kojic acid [179]. Recently, monocerin was isolated from the culture of the entomogenous fungus, *Setosphaeria rostrata* [180].

An insecticidal tetramic acid derivative was found in the culture fluid of *Pyrenophora teres*, a leaf pathogen of barley. When added to artificial diet at a concentration of 100 µg/g, it inhibited the larvae growth in *Spodoptera exigua*, *Helicoverpa virescens*, *D. melanogaster*, and *Trichoplusia ni* [181]. Diplosporin (a substance from the group of γ-pyrons) and chaetoglobosin K (from the group of cytochalasins) isolated from the culture of the corn stem rot pathogen *Stenocarpella maydis* showed moderate insecticidal activity against *S. frugiperda*: a decrease in the growth level by 50–75% at a concentration of 1 mg/g of artificial diet [182].

In the screening natural compounds against the pea aphid *A. pisum*, it was shown that cytochalasin A (from the culture of *Pyrenophora semeniperda* and many other micromycetes), cyclopaldic acid and seiridine (both substances were isolated from the culture of *Seiridium cupressi*) have a noticeable deterrent activity. Maximum aphid mortality was achieved when seiridine was used at a concentration of 0.5‒1 µM [183]. According to Masi et al. (2017) [184], gliotoxin from the *Neosartorya pseudofischeri* culture was more active against first-instar larvae *Ae. aegypti* (LD_50_ 26 µg/mL) than cytochalasin A (LD_50_ 85 µg/mL) but was less effective against female of malaria mosquitoes (LD_50_ 2.8 µg/individual) than fusaric acid (LD_50_ 0.8 µg/individual). Some other phytotoxins (seiridine, sphaeropsidin A, papiracillic acid) isolated from *S. cupressi*, *Diplodia cupressi*, and *Ascochyta agropyrina* cultures, respectively, also demonstrated antifeedant properties against *Ae. aegypti* [185,186].

Fungi of the genus *Alternaria* were shown to have a certain potential as producers of insecticidal metabolites. Some small-spored *Alternaria* spp. are known to cause insect mycoses [187,188]. Some phytophages were found to avoid cabbage leaves infected with *A. brassicicola* [189]. An extract from the mycelium of *A. papavericola* 463-021 (=*Brachycladium papaveris*) caused the death of the vetch aphids *Megoura vicea* at the level of the botanical insecticide NeemAzal^®^ [190]. Extracts from *A. alternata* affected *G. melonella* larvae inhibiting its acetylcholinesterase and also suppressing its innate immunity via reduced the number of hemocytes, lysozyme and phenol oxidase activity [191,192]. Unidentified *A. destruens* metabolites were found to inhibit of *S. litura* α-glucosidase, while exhibiting insecticidal properties with an LD_50_ of about 2 mg/g of feed [193]. Approximately 20% of the extracts from cultures of nine *Alternaria* species showed aphidocidal activity against *M. vicea*. Among them strains of *A. saponariae*, *A. japonica*, *A. tenuissima*, *A. penicillata* and *A. papavericola* were the most prospective producers of insecticidal compounds [194].

Some anti-insectan metabolites produced by *Alternaria* spp. were characterized. Thus, *A. brassicae* produces destruxin B, which has both phytotoxic and insecticidal properties [195,196]. Tenuazonic acid, a phytotoxic mycotoxin of some *Alternaria* species, showed larvicidal activity against the first-instar larvae of *L. sericata* (LD_50_ 120 µg/mL), but was not active against other tested insects and mites from different orders: *D. melanogaster*, *Sitophilus granarius*, *Aphis fabae*, and *T. urticae* [197]. At a concentration of 0.06 µg/mL, which is non-toxic both for China rose *Rosa chinensis* and the rose aphid *Macrosiphum rosivorum*, tenuazonic acid was able to significantly inhibit the reproduction of this insect, which is presumably associated with the induction of repellents release in leaves treated with this toxin [198,199]. At a concentration of 1 mg/mL, methyl-3,8-dihydroxy-6-methyl-4-chloro-9-oxo-9H-xanthene-1-carboxylate and chloromonilinic acid B isolated from *A. sonchi* caused 75% mortality of the aphid *S. graminum* [200]. 

### 2.5. Macromycetes 

It seems that mushrooms (*Basidiomycetes*) do not cause diseases of arthropods. Moreover, their mycelium is nutritious for them. Among arthropods, mycetophages mainly include mites, beetles, and flies. Insects that feed on agarics are mostly polyphagic because the fruit bodies are unpredictable and ephemeral resources [201]. There are macromycetes (for example, members of the genera *Lepista*, *Clitocybe*, and *Cantharellus*) that are rarely affected by insects, and they obviously form and/or can serve as a source of insecticidal substances. Many macrofungi produce poisons and bitter substances (deterrents). Their significance in the life of mushrooms is not precisely established. Perhaps they prevent them from being eaten by invertebrates and vertebrates [202]. The elements of both permanent and damage-induced chemical protection against mycetophages and animals can be found in macromycetes [203,204].

Anti-insectan properties of extracts from the fruit bodies of some macromycetes (*Ganoderma lucidum*, *Pycnoporus sanguinolentus*, *Lactarius gymnocarpoides*, *L. densifolius*, *Russula cellulata*, etc.) are known [205,206,207,208,209]. However, a restricted number of individual insecticidal metabolites have been isolated so far. For instance, oxiran-2-yl-methylpentanoate from the submerged culture of *Cryptotrama asprata* demonstrated high activity against *Ae. aegypti* mosquito larvae at a concentration of about 1.25 µg/g [210].

Some mushrooms were shown to produce protective molecules from non-toxic precursors in response to damage. The usual chemical mechanisms are the hydrolysis of esters, the oxydation of phenols, and the peroxidation of lipids. For example, *Stephanospora caroticolor* forms entomotoxic 2-chloro-4-nitrophenol from stephanosoporin by oxydationas a result of mycelium damage. The mechanical damage to the mycelium of *Aleurodiscus amorphous* induced formation of prussic acid from aleurodisconitrile [203,211]. Two polyenes with antifeedant properties were detected in an unidentified basidiomycete from *Stereaceae*, both accumulated in the damaged mycelium. The gene of polyketide synthase responsible for the biosynthesis of these metabolites was cloned into *Aspergillus niger* genome to perform its heterologous expression and to obtain the corresponding metabolites [212].

## 3. Volatile Organic Compounds (VOCs)

Most publications concerning the volatile metabolites of fungi are devoted to their attractive effect on insects. The fungal smell attracts mycetophages as well as insects that carry on conidia of saprotrophic and phytopathogenic fungi. Thus, insect-attractive VOCs were found in some species of saprotrophic and phytopathogenic basidiomycetes (tinder, rust, smut fungi), ascomycetes (species of the genus *Aspergillus*, *Claviceps*, *Fusarium*) and a number of others. Moreover, some fungal VOCs may be identical to the components of insect pheromones or the odors of flowering plants [213,214,215,216,217].

Significantly less information is available on fungal repellents. The mycelium of various strains of *B. bassiana* had a predominantly repellent effect on the common barn weevil *Sitophilus granaries*, while the mycelium of *Lecanicillium muscarium* had both attractive and repellent properties, depending on the strain of the fungus [218]. Among the approximately one hundred VOCs identified in *B. bassiana*, *Metarhizium robertsii* and *Pochonia chlamydosporia*, 3-cyclohepten-1-one and 1,3-dimethoxybenzene had the maximum repellent activity against the banana weevil *Cosmopolites sordidus*. It is thought that these VOCs could be used in future studies to control this pest in the field [219]. Interestingly, different types of entomopathogenic fungi are characterized by their own set of VOCs, which can be used for chemotaxonomy purposes [220].

The ability to synthesize VOCs with repellent properties was revealed in some endophytic fungi. Thus, *Muscodor vitigenus* isolated from the liana *Paullina paullinioides* forms naphthalene. The fungal culture repelled imago of the wheat stem sawfly *Cephus cinctus* [221]. An unidentified endophyte (strain AP-796) isolated from green foxtail (*Setaria viridis*) produces 3-(4-methylfuran-3-yl)-propane-1-ol that repels the white-spotted stink bug *Eysarcoris ventralis* [222].

## 4. Insecticidal Proteins

Insecticidal proteins of different degrees of characterization have been found in entomopathogenic and phytopathogenic micromycetes, as well as in mushrooms.

Protein extracts from the culture fluid of 25 isolates belonging to *M. anisopliae*, *B. bassiana*, *B. brongniartii*, and *Scopulariopsis brevicaulis* were tested on *Spodoptera littoralis* larvae. Among them, extracts from two isolates of *M. anisopliae* and two isolates of *B. bassiana* were entomotoxic when assayed using leaf disc method or when included in an artificial diet [223]. A selective protein toxin (bassiacridine) with LD_50_ of 3 µg/g of locust *Locusta migratoria* body was isolated from the culture filtrate of *B. bassiana* grown on Adamek medium [224]. Bassiacridine was not studied further. A protein (35.5 kDa) purified from the *B. bassiana* culture was toxic to *G. mellonella* larvae when injected intragemocellularly at a concentration of LD_50_ of 334 µg/g body [225]. In a partially purified protein fraction from the culture liquid *M. anisopliae*, toxic to the fly *C. capitata* at a concentration of 4 mg/mL, four entomotoxic proteins from 11 kDa to 25 kDa were found. The content of toxic proteins in cultures of different *M. anisopliae* strains obtained on different media strongly correlated with their virulence [226,227,228].

A ribotoxin (protein inhibitor of the functioning of ribosomes), hirsutellin A (HtA), was detected in the culture of the entomopathogenic *Hirsutella thompsonii* [229]. Methods for producing HtA using a recombinant strain of *Pichia pastoris* and fermentation in a bioreactor with a yield of 80 mg/L were developed. This allowed a better characterization to be achieved for this protein: its molecular weight (15.3 kDa), stability (high resistance to proteases and temperature), toxicity to Sf9 cells, and insecticidal activity against *G. mellonella* larvae were determined [230]. An analog of HtA, anisoplin, was found in *M. anisopliae* [231].

Agglutinin consisting of two subunits with a molecular weight of 15.5 kDa was isolated from the culture of the *Rhizoctonia solani* strain, which actively forms sclerotia. In the sclerotia of the fungus, its content (at the level of 2–3% of the total soluble protein) was significantly higher than in the mycelium (0.1–2%). A significant reduction in the weight of *S. litura* larvae was observed when lectin was added to the feed at a concentration of 0.1% or higher [232]. From sclerotia of *Sclerotinia sclerotiorum* obtained on a solid natural substrate, another agglutinin was isolated, toxic to pea aphids at a concentration of 1 µg/mL [233].

Proteins were found to be responsible for entomotoxic activity of fruit bodies of 14 species of macromycetes [234]. A serine protease inhibitor (SPI) isolated from *Clitocybe nebularis* inhibited the development of *D. melanogaster* larvae [235]. Sucrose- and lactose-binding lectins from *C. nebularis* fruit bodies showed the activity against larvae of *D. melanogaster* and *L. decemlineata*, respectively [236]. Ribotoxin (135 amino acid residues, ~15 kDa), which has a wide range of biological activity, was isolated from the fruit bodies of *Agrocybe aegerita*. Obtained by heterologous expression in *Escherichia coli*, it demonstrated activity against *Ae. aegypti* larvae [237,238,239].

## 5. Promising Areas of Research

The above data indicate that the most interesting ecological group of fungi for the mining of low-molecular weight compounds with insecticidal properties are soil micromycetes, in particular, from the genera *Aspergillus* and *Penicillium*. However, the biodiversity of soil fungi is obviously underexplored in searching the anti-insectan metabolites. Moreover, the soil as an arena for strong competition between organisms seems to be the specific niche for the producers (both bacteria and fungi) of entomotoxic compounds. However, it is still unclear what type of the soil is favorable for their biodiversity and activity.

Numerous metabolites (especially minor ones) of EPF have not been commonly bioassayed on arthropods [52,90] and still awaited the evaluation for anti-insectan activity. Other groups of fungi, phylogenetically related to soil micromycetes and EPF can be used for screening programs. For instance, the study of insecticidal metabolites of marine species (e.g., *Acremonium*, *Beauveria*, *Penicillium*, etc.) successfully has begun [240,241,242].

Colonization of plants with endophytic fungi was found to decrease a number of some phytophagous insects on the common host [157]. Therefore, endophytes, which are a rich source of bioactive natural compounds, recently attracted renewal interest as producers of insecticidal metabolites [243,244,245]. 

Phytopathogenic fungi (for example, pathogens of cereals and weeds) can also produce anti-insectan compounds [194,200,246,247]. However, they seem structurally similar to those that produce soil and entomopathogenic fungi with a little practical significance but great potential in ecological studies.

It is possible to expand the number of new insecticidal compounds not only by increasing the volume of screening (species and strains of fungi), but also by using various bioassays and types of tested insects [6,248]. However, due to the limited amounts of natural substances obtained from fungal cultures and their high consumption in insect bioassays, it is usually difficult to estimate the spectrum of their insecticidal activity. Perhaps, more sensitive tests on cell cultures, key enzymes from insects could increase the effectiveness of the screening.

A new area of research for the identification of bioactive proteins and secondary metabolites of various organisms is the analysis of their genomes for the presence of genes of secreted proteins and biosynthetic gene clusters (BGC) and their activation using various methods. Several approaches have been tested for the activization of silent BGC, for example, wide variation in the composition of nutrient media, co-cultivation with other microorganisms or insect cells, modification of regulatory proteins, heterologous expression, and others. These studies should be alongside with high-throughput techniques of extraction of fungal metabolites and their analysis by various methods of chromatography and mass spectrometry.

A number of factors (the composition of the nutrient medium, the presence of light, the duration of cultivation) can significantly affect metabolic processes in fungi and affect, for instance, the yield of beauvericin in *B. bassiana* [249,250]. The addition of some amino acids to the liquid culture medium or cultivation of *B. bassiana* on brain tissues of the ant *Camponotus pennsylvanicus* stimulated the production of several new variants of beauveriolides [251,252]. In a mixed culture of *B. bassiana*, the endophyte *Irpex lacteus*, and the phytopathogen *Nigrospora oryzae*, five new compounds were identified, but of the 20 isolated substances, tremulendiol A at a concentration of 6.25 µg/cm^2^ had the maximum toxicity for silkworm [253]. In-depth metabolomic studies using MS/MS and high-resolution mass spectrometry revealed new compounds in *Metarhizium* spp., in particular, previously undescribed destruxins [254].

Genomic analysis of *B. bassiana*, *Cordyceps militaris*, and *M. robertsii* showed a high probability of finding new interesting secondary metabolites and insecticidal proteins. Thus, the genes homologous to the genes of *Bacillus thuringiensis* responsible for the synthesis of protein toxins were found in the genome of *B. bassiana* [255]. The analysis of the genomes of EPFs shows the predominance of unexplored BGC in them, which may indicate high prospects for the detection of previously unknown substances [256]. Interestingly, the analysis of BGC in the genome of *Aspergillus fumigatus* an opportunistic pathogen of humans and insects revealed a large number of unknown BGC, as well as a number of genes responsible for the synthesis of toxins previously identified in *M. robertsii*, as well as phytopathogenic and soil fungi [257]. Heterologous expression of BGC of decalin-containing diterpene pyrones (DDPS) of several entomopathogenic and phytopathogenic fungi and genes responsible for their chemical modification in *Aspergillus oryzae* made it possible to obtain 22 DDPS, of which 15 were new. Some of them (subglutinol A and two new DDPS) showed insecticidal activity against the fruit fly *D. melanogaster* [258].

Along with low-molecular-weight anti-insectan compounds fungi can form protein toxins that can exhibit more specific insecticidal activity. A similar situation is observed for bacteria from the genus *Bacillus*, which produce both insecticidal proteins and a set of other secondary metabolites diverse in the spectrum of biological activity [259,260,261]. The direct use of entomotoxic proteins as biorational insecticides is unlikely due to their instability and low yield. However, the proteins can be used in biotechnology to create genetically modified plants (GMP) that are resistant to phytophagous insects or to construct hypervirulent strains of bioinsecticides (HSB). It may be very interesting to continue earlier studies on the detection of insecticidal proteins of mushrooms [234] and their use along with insecticidal proteins of EPF for development of GMP and HSB. 

Methods for creating GMP containing genes responsible for the expression of plant- incorporated protectants have been successfully developed and are widely used in practice in some countries. Plant varieties carrying *Bacillus thuringiensis* genes for the synthesis of various protein toxins have been commercialized. Enzyme inhibitors, proteases, and lectins are also considered promising insecticidal protectants for the development of new GMP varieties [10].

Approaches for the creation of effective and safe HSB have been underway. For instance, enhanced efficacy of mycoinsecticides can be achieved by adding the copies of virulence factor or stress resistance genes into the genome of biocontrol strains [262]. One of the earliest works in this direction was devoted to embedding an additional copy of the *Pr1* gene encoding the synthesis of a protease, which is involved in the cuticle destruction, into *M. anisopliae* genome. Affected by a transgenic strain, the caterpillars of the tobacco hornworm (*Manduca sexta*) ate less intensively and died 25% faster than after inoculation with wild type strain [263]. Incorporation of the *AaIT* gene, encoding neurotoxic protein of the scorpion *Androctonus australis*, into the *M. anisopliae* genome, demonstrated its higher virulence for the tobacco hornworm with 22-fold decrease in LD_50_ compared to the action of wild type strain [264]. Similar work was done later for other EPF: *Lecanicillium lecanii* [265] and *B. bassiana* [266]. The production of the recombinant toxin Bt-Vip3A of *B. thuringiensis* in a genetically modified strain of *B. bassiana* significantly increased its virulence against *S. litura* larvae and led to a 15‒26-fold decrease in LD_50_ [267]. The expression of another bacterial protein Cyt2Ba in *B. bassiana* increased its biocontrol effectiveness against two mosquito species, *Ae. aegypti* and *Ae. albopictus* [268].

Obviously, such genetic engineering studies to create HSB to control harmful arthropods have great prospects. However, the lack of permission for the use of transgenic microorganisms in agriculture in most countries of the world and the lack of information about their safety delay their practical use. Nevertheless, the development of these technologies can be very useful in the future [9].

## 6. Conclusions

Despite the fact that the chemical method remains prevalent in practice due to the relative ease of production and application, higher biological efficiency and stability, there is a clear trend towards an increase in the number of registered insecticides of natural origin. Fungi, along with bacteria and plants, are a source of new anti-insectan molecules, some of which have already been used to develop commercial insecticides against pest arthropods.

As this review demonstrates, the potential of fungi as producers of substances with insecticidal, antifeedant and hormonal properties is far from being exhausted. The analysis of the literature showed that soil micromycetes are the most promising ecological group of fungi for their search. Metabolites with insecticidal and antifeedant effects were also found in endophytic and phytopathogenic fungi. The main metabolites of entomopathogenic fungi are mainly immunosuppressants with moderate insecticidal properties. Mushrooms is a promising group for the production of antifeedant compounds and insecticidal proteins. The chemical ecology approach is hoped to be useful to reveal new fungal taxa (or ecological groups) as producers of anti-insectan compounds.

It is possible to expand the number of substances with insecticidal properties detected in fungi not only by increasing the volume of screening, but also by using various bioassays and types of tested insects. The analysis of fungal genomes for the presence of genes of secreted proteins and clusters of genes of secondary metabolites with their subsequent activation by various methods is also promising. To increase the efficiency of these works, it is necessary to use high-throughput methods of extraction of metabolites for fungal cultures and their analysis by various methods of chromatography and mass spectrometry. Insecticidal proteins identified in fungi can be used in the future in technologies for creating transgenic plant varieties that are resistant to pests, or hypervirulent bioinsecticides.

However, the detection of substances that affect insects is only the first stage in the development of new biorational insecticides. In the next stages of this work, their extensive toxicological studies and determination of action mechanisms will be required. It may be followed by the development of approaches to enhance their efficacy, increasing the yield in culture (selection of media and cultivation conditions, metabolic engineering of producing strains), obtaining more active synthetic derivatives or analogues, improving the formulations (for example, formulations with controlled release for relatively toxic compounds) and methods of application (for example, studying the effects of synergism or sensitization when used with other insecticides).

## Figures and Tables

**Table 1:** Ways of using various natural compounds for control of pest arthropods.

Group of Natural Compounds	Possible Use
Low molecular weight non-volatile compounds	Insecticides, antifeedants, and insect growth regulators
	Synthesis of more effective analogues or semi-synthetic derivatives of natural compounds
Volatile organic compounds	Biofumigants, repellents, attractants
Insecticidal proteins	Development of transgenic hypervirulent bioinsecticides
	Development of transgenic plants resistant to pests

**Table 2:** Examples of insecticides based on natural compounds *.

Trade Name	Active Ingredients	Producer	Manufacturer
Fitoverm^®^	Aversektin С (a mixture of avermectins)	*Streptomyces avermitilis*	Farmbiomed, Russia
Vertimec^®^	Abamektin (a mixture of avermectins В1а and В1b)		Syngenta, Switzerland
Spintor^®^	Spinosade (a mixture of spinosins A and D)	*Saccharopolyspora spinosa*	Corteva, USA
NeemAzal^®^	Azadirachtin	*Azadirachta indica*	Trifolio-M GmbH, Germany
Requiem^®^	A blend of α-terpinene, ρ-cymene, d-limonene	*Chenopodium mbrosioides*	Bayer, Germany
FLiPPER^®^	Long chain unsaturated carboxylic acids derived from olive oil	*Olea europaea*	Bayer, Germany
Inscalis^®^	Afidopyropen, a semisynthetic product of pyripyropen A	*Penicillium coprobium*	BASF, Germany

* Based on data from open online resources.

**Table 3:** Fungal metabolites with antagonistic action against arthropods.

Type of Activity	Effect	Expected Result
Insecticides	Entomotoxicity	Death
Mycotoxins	Non-selective toxicity	
Immuno-suppressant	Increased susceptibility to entomopathogens and opportunistic infections	Increased mortality
Antibiotics	Inhibition of intestinal microflora, suppression of immunity	
Repellents, deterrents	Deterioration of the substrate quality scaring off due to “unpleasant” smell or taste	
Elicitors/effectors	Deterioration of the substrate quality due to toxic plant metabolites or attraction of entomophagous insects	Slow development, reduced fertility
Phytotoxins	Deterioration of the substrate quality due death of plant cells	

## References

[1] Zehnder G., Gurr G.M., Kühne S., Wade M.R., Wratten S., Wyss E. (2007). Arthropod Pest Management in Organic Crops. Annu. Rev. Èntomol..

[2] Rebek E.J., Frank S.D., Royer T.A., Bográn C.E. (2012). Alternatives to Chemical Control of Insect Pests. Insecticides—Basic and Other Applications.

[3] Rosell G., Quero C., Coll J., Guerrero A. (2008). Biorational insecticides in pest management. J. Pestic. Sci..

[4] Horowitz A.R., Ellsworth P.C., Ishaaya I. (2009). Biorational Pest Control—An Overview.

[5] Haddi K., Turchen L.M., Jumbo L.O.V., Guedes R.N.C., Pereira E.J.G., Aguiar R.W.S., Oliveira E.E. (2020). Rethinking biorational insecticides for pest management: Unintended effects and consequences. Pest Manag. Sci..

[6] Yu S.J. (2014). The toxicology and biochemistry of insecticides.

[7] Smagghe G., Zotti M., Retnakaran A. (2019). Targeting female reproduction in insects with biorational insecticides for pest management: A critical review with suggestions for future research. Curr. Opin. Insect Sci..

[8] Isman M. (2002). Insect antifeedants. Pestic. Outlook.

[9] Lovett B., Leger R.J.S. (2018). Genetically engineering better fungal biopesticides. Pest Manag. Sci..

[10] Nelson M.E., Alves A.P. (2014). Plant Incorporated Protectants and Insect Resistance. Insect Resistance Manag..

[11] Turchen L.M., Júnior L.C., Guedes R.N.C. (2020). Plant-Derived Insecticides Under Meta-Analyses: Status, Biases, and Knowledge Gaps. Insects.

[12] Jeschke P. (2021). Status and outlook for acaricide and insecticide discovery. Pest Manag. Sci..

[13] Bills G.F., Gloer J.B. (2017). Biologically Active Secondary Metabolites from the Fungi. Fungal Kingd..

[14] Karwehl S., Stadler M. (2016). Exploitation of Fungal Biodiversity for Discovery of Novel Antibiotics. Curr. Top. Microbiol. Immunol..

[15] Letten A.D., Hall A.R., Levine J.M. (2021). Using ecological coexistence theory to understand antibiotic resistance and microbial competition. Nat. Ecol. Evol..

[16] Spiteller P. (2015). Chemical ecology of fungi. Nat. Prod. Rep..

[17] Boucias D.G., Lietze V.U., Teal P. (2012). Chemical Signals That Mediate Insect-Fungal Interactions. Biocommunication of Fungi.

[18] Beemelmanns C., Guo H., Rischer M., Poulsen M. (2016). Natural products from microbes associated with insects. Beilstein J. Org. Chem..

[19] Grunseich J.M., Thompson M.N., Aguirre N., Helms A.M. (2019). The Role of Plant-Associated Microbes in Mediating Host-Plant Selection by Insect Herbivores. Plants.

[20] Francis F., Jacquemyn H., Delvigne F., Lievens B. (2020). From Diverse Origins to Specific Targets: Role of Microorganisms in Indirect Pest Biological Control. Insects.

[21] Dowd P.F., Koul O., Dhaliwal G.S. (2002). Antiinsectan compounds derived from microorganisms. Microbial Biopesticides.

[22] Holighaus G., Rohlfs M. (2016). Fungal allelochemicals in insect pest management. Appl. Microbiol. Biotechnol..

[23] Lacey L. (2017). Entomopathogens Used as Microbial Control Agents. Microbial Control of Insect and Mite Pests.

[24] Butt T., Coates C., Dubovskiy I., Ratcliffe N. (2016). Entomopathogenic Fungi: New Insights into Host-Pathogen Interactions. Fungal Genomics.

[25] Wang C., Wang S. (2017). Insect Pathogenic Fungi: Genomics, Molecular Interactions, and Genetic Improvements. Annu. Rev. Èntomol..

[26] Oller-López J.L., Iranzo M., Mormeneo S., Oliver E., Cuerva J.M., Oltra J.E. (2005). Bassianolone: An antimicrobial precursor of cephalosporolides E and F from the entomoparasitic fungus Beauveria bassiana. Org. Biomol. Chem..

[27] Zimmermann G. (2007). Review on safety of the entomopathogenic fungi Beauveria bassiana and Beauveria brongniartii. Biocontrol Sci. Technol..

[28] Neumann K., Kehraus S., Gütschow M., König G.M. (2009). Cytotoxic and HLE-Inhibitory Tetramic Acid Derivatives from Marine-Derived Fungi. Nat. Prod. Commun..

[29] Song L., Lee K.H., Lin Z., Tong R. (2014). Structural Revision of Cephalosporolide J and Bassianolone. J. Org. Chem..

[30] Vey A., Hoagland R.E., Butt T.M. (2009). Toxic metabolites of fungal biocontrol agents. Fungi as Biocontrol Agents: Progress, Problems and Potential.

[31] Uchida R., Imasato R., Yamaguchi Y., Masuma R., Shiomi K., Tomoda H., Omura S. (2005). New Insecticidal Antibiotics, Hydroxyfungerins A and B, Produced by Metarhizium sp. FKI-1079. J. Antibiot..

[32] Zimmermann G. (2007). Review on safety of the entomopathogenic fungus Metarhizium anisopliae. Biocontrol. Sci. Technol..

[33] Xu Y.-J., Luo F., Li B., Shang Y., Wang C. (2016). Metabolic Conservation and Diversification of Metarhizium Species Correlate with Fungal Host-Specificity. Front. Microbiol..

[34] Leadmon C.E., Sampson J.K., Maust M.D., Macias A.M., Rehner S.A., Kasson M.T., Panaccione D.G. (2020). Several Metarhizium Species Produce Ergot Alkaloids in a Condition-Specific Manner. Appl. Environ. Microbiol..

[35] Prathumpai W., Kocharin K. (2016). Phomalactone optimization and production of entomopathogenic fungi by Ophiocordyceps communisBCC 1842 and BCC 2763. Prep. Biochem. Biotechnol..

[36] Wang J., Zhang D.-M., Jia J.-F., Peng Q.-L., Tian H.-Y., Wang L., Ye W.-C. (2014). Cyclodepsipeptides from the ascocarps and insect-body portions of fungus Cordyceps cicadae. Fitoterapia.

[37] Luangsa-Ard J.J., Berkaew P., Ridkaew R., Hywel-Jones N.L., Isaka M. (2009). A beauvericin hot spot in the genus Isaria. Mycol. Res..

[38] Zhan J., Burns A.M., Liu M.X., Faeth S.H., Gunatilaka A.A.L. (2007). Search for Cell Motility and Angiogenesis Inhibitors with Potential Anticancer Activity: Beauvericin and Other Constituents of Two Endophytic Strains ofFusarium oxysporum1. J. Nat. Prod..

[39] Vikhe A. (2016). In Vitro and In Vivo Induction, and Characterization of Toxins Isolated from Beauveria bassiana. Int. J. Pure Appl. Biosci..

[40] Grove J.F., Pople M. (1980). The insecticidal activity of beauvericin and the enniatin complex. Mycopathol..

[41] Ganassi S., Moretti A., Pagliai A.M.B., Logrieco A.F., Sabatini M.A. (2002). Effects of beauvericin on Schizaphis graminum (Aphididae). J. Invertebr. Pathol..

[42] Gupta S., Montllor C., Hwang Y.-S. (1995). Isolation of Novel Beauvericin Analogues from the Fungus Beauveria bassiana. J. Nat. Prod..

[43] Fukuda T., Arai M., Yamaguchi Y., Masuma R., Tomoda H., Omura S. (2004). New beauvericins, potentiators of antifungal miconazole activity, Produced by Beauveria sp. FKI-1366. I. Taxonomy, fermentation, isolation and biological properties. J. Antibiot..

[44] Al Khoury C., Guillot J., Nemer N. (2019). Lethal activity of beauvericin, a Beauveria bassiana mycotoxin, against the two-spotted spider mites, Tetranychus urticaeKoch. J. Appl. Èntomol..

[45] Valencia J.W.A., Bustamante A.L.G., Jiménez A.V., Grossi-De-Sa M.F. (2011). Cytotoxic Activity of Fungal Metabolites from the Pathogenic Fungus Beauveria bassiana: An Intraspecific Evaluation of Beauvericin Production. Curr. Microbiol..

[46] Fornelli F., Minervini F., Logrieco A.F. (2004). Cytotoxicity of fungal metabolites to lepidopteran (Spodoptera frugiperda) cell line (SF-9). J. Invertebr. Pathol..

[47] Wang Q., Xu L. (2012). Beauvericin, a Bioactive Compound Produced by Fungi: A Short Review. Molecules.

[48] Bharani R.S.A., Namasivayam S.K.R., Shankar S.S. (2014). Biocompatible chitosan nanoparticles incorporated pesticidal protein beauvericin (Csnp-Bv) preparation for the improved pesticidal activity against major groundnut defoliator Spodoptera litura (Fab.) (Lepidoptera; Noctuidae). Int. J. Chemtech. Res..

[49] Urbaniak M., Waśkiewicz A., Stępień Ł. (2020). *Fusarium* Cyclodepsipeptide Mycotoxins: Chemistry, Biosynthesis, and Occurrence. Toxins.

[50] Mallebrera B., Prosperini A., Font G., Ruiz M.J. (2018). In vitro mechanisms of Beauvericin toxicity: A review. Food Chem. Toxicol..

[51] Šrobárová A., Da Silva J.A.T., Kogan G., Ritieni A., Santini A. (2009). Beauvericin Decreases Cell Viability of Wheat. Chem. Biodivers..

[52] Yin Y., Chen B., Song S., Li B., Yang X., Wang C. (2020). Production of Diverse Beauveriolide Analogs in Closely Related Fungi: A Rare Case of Fungal Chemodiversity. mSphere.

[53] Mochizuki K., Ohmori K., Tamura H., Shizuri Y., Nishiyama S., Miyoshi E., Yamamura S. (1993). The Structures of Bioactive Cyclodepsipeptides, Beauveriolides I and II, Metabolites of Entomopathogenic FungiBeauveriasp. Bull. Chem. Soc. Jpn..

[54] Vilcinskas A., Jegorov A., Landa Z., Götz P., Matha V. (1999). Effects of beauverolide L and cyclosporin A on humoral and cellular immune response of the greater wax moth, Galleria mellonella. Comp. Biochem. Physiol. Part C Pharmacol. Toxicol. Endocrinol..

[55] El Basyouni S.H., Brewer D., Vining L.C. (1968). Pigments of the genus Beauveria. Can. J. Bot..

[56] Strasser H., Abendstein D., Stuppner H., Butt T. (2000). Monitoring the distribution of secondary metabolites produced by the entomogenous fungus Beauveria brongniartii with particular reference to oosporein. Mycol. Res..

[57] Eyal J., Fischbein K.L., Walter J.F. (1993). Novel toxin producing fungal pathogen and uses. European Patent.

[58] Amin G.A., Youssef N.A., Bazaid S., Saleh W.D. (2010). Assessment of insecticidal activity of red pigment produced by the fungus Beauveria bassiana. World J. Microbiol. Biotechnol..

[59] Spiridonov N., Arkhipov V., Narimanov A., Shabalina S., Zverkova L., Shvirst E., Kondrashova M. (1992). Effect of Galleria mellonella larvae preparation and honeybee products on cell cultures. Comp. Biochem. Physiol. Part C Comp. Pharmacol..

[60] Nagaoka T., Nakatab K., Kouno K. (2004). Antifungal Activity of Oosporein from an Antagonistic Fungus against Phytophthora infestans. Zeitschrift für Naturforschung C.

[61] Alurappa R., Bojegowda M.R.M., Kumar V., Mallesh N.K., Chowdappa S. (2014). Characterisation and bioactivity of oosporein produced by endophytic fungus *Cochliobolus kusanoi* isolated from *Nerium oleander* L.. Nat. Prod. Res..

[62] Feng P., Shang Y., Cen K., Wang C. (2015). Fungal biosynthesis of the bibenzoquinone oosporein to evade insect immunity. Proc. Natl. Acad. Sci. USA.

[63] Fan Y., Liu X., Keyhani N.O., Tang G., Pei Y., Zhang W., Tong S. (2017). Regulatory cascade and biological activity of Beauveria bassiana oosporein that limits bacterial growth after host death. Proc. Natl. Acad. Sci. USA.

[64] Lozano-Tovar M.D., Garrido-Jurado I., Lafont F., Quesada-Moraga E. (2015). Insecticidal Activity of a Destruxin-Containing Extract of Metarhizium brunneum Against Ceratitis capitata (Diptera: Tephritidae). J. Econ. Èntomol..

[65] Sabbour M.M. (2019). Effect of destruxin on the population reduction of green peach aphid Myzus persicae (Hemiptera: Aphididae) and the predator Coccinella undecimpunctata (Coleoptera: Coccinellidae) in tomato fields. Bull. Natl. Res. Cent..

[66] Sabbour M.M., Solieman N.Y., Abdel-Raheem M.A. (2020). Influence of Metarhizium anisopliae-based destruxin A-760 and destruxin—A-724 on the sugar beet fly, Pegomya mixta Vill. (Diptera: Anthomyiidae). J. Biopestic..

[67] Pal S., Leger R.J.S., Wu L. (2007). Fungal Peptide Destruxin A Plays a Specific Role in Suppressing the Innate Immune Response in Drosophila melanogaster. J. Biol. Chem..

[68] Hu W., He G., Wang J., Hu Q. (2016). The Effects of Destruxin A on Relish and Rel Gene Regulation to the Suspected Immune-Related Genes of Silkworm. Molecules.

[69] Fan J.-Q., Chen X.-R., Hu Q.-B. (2013). Effects of Destruxin A on Hemocytes Morphology of Bombyx mori. J. Integr. Agric..

[70] Wang J., Weng Q., Yin F., Hu Q. (2020). Interactions of Destruxin A with Silkworms’ Arginine tRNA Synthetase and Lamin-C Proteins. Toxins.

[71] Feng K.-C., Rou T.-M., Liu B.-L., Tzeng Y.-M., Chang Y.-N. (2004). Effect of fungal pellet size on the high yield production of destruxin B by Metarhizium anisopliae. Enzym. Microb. Technol..

[72] Hu Q.-B., Ren S.-X., Wu J.-H., Chang J.-M., Musa P.D. (2006). Investigation of destruxin A and B from 80 Metarhizium strains in China, and the optimization of cultural conditions for the strain MaQ10. Toxicon.

[73] Kim H.M., Choi I.S., Lee S., Hwang I.M., Chun H.H., Wi S.G., Kim J.-C., Shin T.Y., Kim J.C., Kim J.S. (2019). Advanced strategy to produce insecticidal destruxins from lignocellulosic biomass Miscanthus. Biotechnol. Biofuels.

[74] Gupta S., Krasnoff S.B., Renwick J.A.A., Roberts D.W., Steiner J.R., Clardy J. (1993). Viridoxins A and B: Novel toxins from the fungus Metarhizium flavoviride. J. Org. Chem..

[75] Tuli H.S., Sharma A.K., Sandhu S.S., Kashyap D. (2013). Cordycepin: A bioactive metabolite with therapeutic potential. Life Sci..

[76] Holliday J.C., Cleaver M.P. (2008). Medicinal Value of the Caterpillar Fungi Species of the Genus Cordyceps (Fr.) Link (Ascomycetes): A Review. Int. J. Med. Mushrooms.

[77] Roberts D.W., Burges H.D. (1981). Toxins of entomopathogenic fungi. Microbial Control of Pests and Plant Disease 1970–1980.

[78] Kim J.-R., Yeon S.-H., Kim H.-S., Ahn Y.-J. (2002). Larvicidal activity against Plutella xylostella of cordycepin from the fruiting body ofCordyceps militaris. Pest Manag. Sci..

[79] Tyurin M.V., Orlova T.I., Tomilova O.G., Saveleva E.I., Kryukov V.Y., Berestetskiy A.O. (2019). Production of Cordyceps militaris metabolites in infected wax moth Galleria mellonella L. larvae. Euroasian Èntomol. J..

[80] Woolley V.C., Teakle G.R., Prince G., de Moor C.H., Chandler D. (2020). Cordycepin, a metabolite of Cordyceps militaris, reduces immune-related gene expression in insects. J. Invertebr. Pathol..

[81] Quy T.N., Xuan T.D., Andriana Y., Tran H.-D., Khanh T.D., Teschke R., Qu X. (2019). Tran Cordycepin Isolated from Cordyceps militaris: Its Newly Discovered Herbicidal Property and Potential Plant-Based Novel Alternative to Glyphosate. Molecules.

[82] Chen B.-X., Wei T., Xue L.-N., Zheng Q.-W., Ye Z.-W., Zou Y., Yang Y., Yun F., Guo L.-Q., Lin J.-F. (2020). Transcriptome Analysis Reveals the Flexibility of Cordycepin Network in Cordyceps militaris Activated by L-Alanine Addition. Front. Microbiol..

[83] Krasnoff S.B., Gupta S. (1994). Identification of the antibiotic phomalactone from the entomopathogenic fungus Hirsutella thompsonii var. synnematosa. J. Chem. Ecol..

[84] Meepagala K.M., Becnel J.J., Estep A. (2015). Phomalactone as the Active Constituent against Mosquitoes from Nigrospora spherica. Agric. Sci..

[85] Kim J.-C., Choi G.J., Park J.-H., Kim H.T., Cho K.Y. (2001). Activity against plant pathogenic fungi of phomalactone isolated fromNigrospora sphaerica. Pest Manag. Sci..

[86] Borel J.F. (2002). History of the discovery of cyclosporin and of its early pharmacological development. Wien. Klin. Wochenschr..

[87] Yang X., Feng P., Yin Y., Bushley K., Spatafora J.W., Wang C. (2018). Cyclosporine Biosynthesis in Tolypocladium inflatum Benefits Fungal Adaptation to the Environment. mBio.

[88] Dong S., Kang S., Dimopoulos G. (2019). Identification of anti-flaviviral drugs with mosquitocidal and anti-Zika virus activity in Aedes aegypti. PLOS Neglected Trop. Dis..

[89] Fiolka M.J. (2008). Immunosuppressive effect of cyclosporin A on insect humoral immune response. J. Invertebr. Pathol..

[90] Zhang L., Fasoyin O.E., Molnár I., Xu Y. (2020). Secondary metabolites from hypocrealean entomopathogenic fungi: Novel bioactive compounds. Nat. Prod. Rep..

[91] Liao C., Zheng M., Chen Y., Wang M., Li B. (2019). Immunosuppression mechanism of entomopathogenic bacteria against Galleria mellonella larvae. Process. Biochem..

[92] Roy M.C., Lee D., Kim Y. (2019). Host Immunosuppression Induced by Steinernema feltiae, an Entomopathogenic Nematode, through Inhibition of Eicosanoid Biosynthesis. Insects.

[93] Pietri J.E., Liang D. (2020). Insecticidal Activity of Doxycycline against the Common Bedbug. Antimicrob. Agents Chemother..

[94] Ishidoh K.-I., Kinoshita H., Igarashi Y., Ihara F., Nihira T. (2014). Cyclic lipodepsipeptides verlamelin A and B, isolated from entomopathogenic fungus *Lecanicillium* sp.. J. Antibiot..

[95] Wagenaar M.M., Gibson D.M., Clardy J. (2002). Akanthomycin, a New Antibiotic Pyridone from the Entomopathogenic FungusAkanthomycesgracilis. Org. Lett..

[96] Bunbamrung N., Intaraudom C., Supothina S., Komwijit S., Pittayakhajonwut P. (2015). Antibacterial and anti-phytopathogenic substances from the insect pathogenic fungus Gibellula sp. BCC36964. Phytochem. Lett..

[97] Dowd P.F. (1992). Insect fungal symbionts: A promising source of detoxifying enzymes. J. Ind. Microbiol. Biotechnol..

[98] Zhou F., Xu L., Wu X., Zhao X., Liu M., Zhang X. (2020). Symbiotic Bacterium-Derived Organic Acids Protect Delia antiqua Larvae from Entomopathogenic Fungal Infection. mSystems.

[99] Woo R.M., Park M.G., Choi J.Y., Park D.H., Kim J.Y., Wang M., Kim H.J., Woo S.D., Kim J.S., Je Y.H. (2020). Insecticidal and insect growth regulatory activities of secondary metabolites from entomopathogenic fungi, Lecanicillium attenuatum. J. Appl. Èntomol..

[100] St Leger R.J., Screen S.E., Shams-Pirzadeh B. (2000). Lack of host specialization in Aspergillus flavus. Appl. Environ. Microbiol..

[101] Ganassi S., Moretti A., Stornelli C., Fratello B., Pagliai A.B., Logrieco A.F., Sabatini M. (2001). Effect of Fusarium, Paecilomyces and Trichoderma formulations against aphid Schizaphis graminum. Mycopathologia.

[102] Abdul-Wahid O.A. (2012). Evaluation of the insecticidal activity of Fusarium solani and Trichoderma harzianum against cockroaches; Periplaneta Americana. Afr. J. Microbiol. Res..

[103] Demirözer O., Uzun A., Arici Ş.-E., Gep I., Bakay R. (2016). Insecticidal effect of Fusarium subglutinans on Frankliniella occidentalis (Pergande) (Thysanoptera: Thripidae). Hell. Plant Prot. J..

[104] Ahmad F., Anwar W., Javed M.A., Basit R., Akhter A., Ali S., AKhan H.A., Amin H., Haider M.S. (2019). Infection mechanism of Aspergillus and Fusarium species against Bemisia tabaci. Mycopath.

[105] Calvo A.M., Cary J.W. (2015). Association of fungal secondary metabolism and sclerotial biology. Front. Microbiol..

[106] Rohlfs M. (2015). Fungal secondary metabolite dynamics in fungus–Grazer interactions: Novel insights and unanswered questions. Front. Microbiol..

[107] Reeves E.P., Messina C., Doyle S., Kavanagh K. (2004). Correlation between gliotoxin production and virulence of Aspergillus fumigatus in Galleria mellonella. Mycopathology.

[108] Fallon J.P., Reeves E.P., Kavanagh K. (2011). The Aspergillus fumigatus toxin fumagillin suppresses the immune response of Galleria mellonella larvae by inhibiting the action of haemocytes. Microbiology.

[109] Panaccione D.G., Arnold S.L. (2017). Ergot alkaloids contribute to virulence in an insect model of invasive aspergillosis. Sci. Rep..

[110] Gloer J.B. (1995). Antiinsectan natural products from fungal sclerotia. Accounts Chem. Res..

[111] Wicklow D.T., Dowd P.F., Alfatafta A.A., Gloer J.B. (1996). Ochratoxin A: An antiinsectan metabolite from the sclerotia of Aspergillus carbonarius NRRL 369. Can. J. Microbiol..

[112] Staub G.M., Dowd P.F., Gloer J.B., Wicklow D.T. (1992). Aspernomine, an Antiinsectan Metabolite. U.S. Patent.

[113] Laakso J.A., TePaske M.R., Dowd P.F., Gloer J.B., Wicklow D.T. (1992). Sulpinine, Secopenitrem B and Aflatrem B Antiinsectan Metabolites. U.S. Patent.

[114] Dowd P.F., Wicklow D.T., Gloer J.B., TePaske M. (1994). Leporin A, an Antiinsectan Fungal Metabolite. U.S. Patent.

[115] Deguzman F.S., Dowd P.F., Gloer J.B., Wicklow D.T. (1994). *N*-Methylepiamauromine, Epiamauromine and Cycloechinulin Antiinsectan Metabolites. U.S. Patent.

[116] Frisvad J., Petersen L.M., Lyhne E.K., Larsen T.O. (2014). Formation of Sclerotia and Production of Indoloterpenes by Aspergillus niger and Other Species in Section Nigri. PLoS ONE.

[117] Petersen L.M., Frisvad J.C., Knudsen P.B., Rohlfs M., Gotfredsen C.H., Larsen T.O. (2015). Induced sclerotium formation exposes new bioactive metabolites from Aspergillus sclerotiicarbonarius. J. Antibiot..

[118] Hayashi H., Nishimoto Y., Akiyama K., Nozaki H. (2000). New Paralytic Alkaloids, Asperparalines A, B and C, fromAspergillus japonicusJV-23. Biosci. Biotechnol. Biochem..

[119] Hirata K., Kataoka S., Furutani S., Hayashi H., Matsuda K. (2011). A Fungal Metabolite Asperparaline A Strongly and Selectively Blocks Insect Nicotinic Acetylcholine Receptors: The First Report on the Mode of Action. PLoS ONE.

[120] Wang H.J., Gloer J.B., Wicklow D.T., Dowd P.F. (1995). Aflavinines and other antiinsectan metabolites from the ascostromata of Eupenicillium crustaceum and related species. Appl. Environ. Microbiol..

[121] González-Mas M.C., Lull C., Moya P., Ayala I., Primo J., Yúfera E.P. (2003). Insecticidal Activity of Penitrems, Including Penitrem G, a New Member of the Family Isolated from Penicillium crustosum. J. Agric. Food Chem..

[122] Omura S., Tomoda H., Kim Y.K., Nishida H. (1993). Pyripyropenes, highly potent inhibitiors of Acyl-CoA:Cholesterol acyltransferase produced by Aspergillus fumigatus. J. Antibiot..

[123] Koradin C., Schröder H., Oyama K., Ōmura S. (2021). Chemistry and biology connected: The development of Inscalis. Recent Highlights in the Discovery and Optimization of Crop Protection Products.

[124] Castillo M.-A., Moya P., Cantín A., Miranda M.A., Primo J., Hernández E., Primo-Yúfera E. (1999). Insecticidal, anti-juvenile hormone, and fungicidal activities of organic extracts from different Penicillium species and their isolated active components. J. Agric. Food Chem..

[125] Cantín Á., Moya P., Castillo M.-A., Primo J., Miranda M.A., Primo-Yúfera E. (1998). Isolation and Synthesis ofN-(2-Methyl-3-oxodec-8-enoyl)-2-pyrroline and 2-(Hept-5-enyl)-3-methyl-4-oxo-6,7,8,8a-tetrahydro-4H-pyrrolo[2,1-b]1,3-oxazine—Two New Fungal Metabolites with in vivo Anti-Juvenile-Hormone and Insecticidal Activity. Eur. J. Org. Chem..

[126] Moya P., Cantín Á., Miranda M.A., Primo J., Primo-Yúfera E. (1999). Synthesis and Biological Evaluation of New Analogues of the Active Fungal MetabolitesN-(2-Methyl-3-oxodecanoyl)-2-pyrroline andN-(2-Methyl-3-oxodec-8-enoyl)-2-pyrroline. J. Agric. Food Chem..

[127] Nielsen M.T., Klejnstrup M.L., Rohlfs M., Anyaogu D.C., Nielsen J.B., Gotfredsen C.H., Andersen M.R., Hansen B.G., Mortensen U.H., Larsen T.O. (2013). Aspergillus nidulans Synthesize Insect Juvenile Hormones upon Expression of a Heterologous Regulatory Protein and in Response to Grazing by Drosophila melanogaster Larvae. PLoS ONE.

[128] Reino J.L., Guerrero R.F., Hernández-Galán R., Collado I.G. (2008). Secondary metabolites from species of the biocontrol agent Trichoderma. Phytochem. Rev..

[129] Contreras-Cornejo H.A., Macías-Rodríguez L., Del-Val E., Larsen J. (2016). Ecological functions of Trichoderma spp. and their secondary metabolites in the rhizosphere: Interactions with plants. FEMS Microbiol. Ecol..

[130] Ganassi S., De Cristofaro A., Grazioso P., Altomare C., Logrieco A.F., Sabatini M.A. (2007). Detection of fungal metabolites of various Trichoderma species by the aphid Schizaphis graminum. Èntomol. Exp. Appl..

[131] Evidente A., Ricciardiello G., Andolfi A., Sabatini M.A., Ganassi S., Altomare C., Favilla M., Melck D. (2008). Citrantifidiene and Citrantifidiol: Bioactive Metabolites Produced by Trichoderma citrinoviride with Potential Antifeedant Activity toward Aphids. J. Agric. Food Chem..

[132] Evidente A., Andolfi A., Cimmino A., Ganassi S., Altomare C., Favilla M., De Cristofaro A., Vitagliano S., Sabatini M.A. (2009). Bisorbicillinoids Produced by the Fungus Trichoderma citrinoviride Affect Feeding Preference of the Aphid Schizaphis graminum. J. Chem. Ecol..

[133] Sabatini M.A., Ganassi S., Altomare C., Favilla M., Evidente A., Andolfi A. (2012). Phagodeterrent Compounds of Fungal Origin. International Patent.

[134] Ganassi S., Grazioso P., De Cristofaro A., Fiorentini F., Sabatini M.A., Evidente A., Altomare C. (2016). Long Chain Alcohols Produced by Trichoderma citrinoviride Have Phagodeterrent Activity against the Bird Cherry-Oat Aphid Rhopalosiphum padi. Front. Microbiol..

[135] Wicklow D.T., Dowd P.F., Gloer J.B. (1999). Chaetomium Mycotoxins with Antiinsectan or Antifungal Activity. Mycotoxins.

[136] Teetor-Barsch G.H., Roberts D.W. (1983). Entomogenous Fusarium species. Mycopatрologia.

[137] Claydon N., Grove J.F., Pople M. (1979). Insecticidal secondary metabolic products from the entomogenous fungus Fusarium larvarum. J. Invertebr. Pathol..

[138] Usseglio V.L., Dambolena J.S., Martinez M.J., Zunino M.P. (2020). The Role of Fumonisins in the Biological Interaction between Fusarium verticillioides and Sitophilus zeamais. J. Chem. Ecol..

[139] Dowd P.F., Burmeister H.R., Vesonder R.F. (1992). Antiinsectan properties of a novel Fusarium-derived sterol sulfate. Èntomol. Exp. Appl..

[140] Reiss J. (1975). Insecticidal and larvicidal activities of the mycotoxins aflatoxin B1, rubratoxin B, patulin and diacetoxyscirpenol towards Drosophila melanogaster. Chem. Interact..

[141] Wright V.F., Harein P.K., Casas E.D.L. (1980). Evaluation of Penicillium Mycotoxins for Activity in Stored-product Coleoptera 1. Environ. Èntomol..

[142] Dowd P.F. (1989). Toxicity of Naturally Occurring Levels of the Penicillium Mycotoxins Citrinin, Ochratoxin A, and Penicillic Acid to the Corn Earworm, Heliothis zea, and the Fall Armyworm, Spodoptera frugiperda (Lepidoptera: Noctuidae). Environ. Èntomol..

[143] Niu G., Johnson R., Berenbaum M.R. (2011). Toxicity of mycotoxins to honeybees and its amelioration by propolis. Apidologie.

[144] Stepanycheva E.A., Petrova M.O., CHermenskaya T.D., Gavrilova O.P., Gagkaeva T.Y.U., Shamshev I.V. (2016). Ecological and biochemical interactions of fungi of the genus Fusarium and phytophages of cereal crops. Evraziatskij Èntomol. Zhurnal.

[145] Drakulic J., Ajigboye O., Swarup R., Bruce T., Ray R.V. (2016). Aphid Infestation Increases Fusarium langsethiae and T-2 and HT-2 Mycotoxins in Wheat. Appl. Environ. Microbiol..

[146] Niu G., Siegel J., Schuler M.A., Berenbaum M.R. (2009). Comparative Toxicity of Mycotoxins to Navel Orangeworm (Amyelois transitella) and Corn Earworm (Helicoverpa zea). J. Chem. Ecol..

[147] Guo Z., Döll K., Dastjerdi R., Karlovsky P., Dehne H.-W., Altincicek B. (2014). Effect of Fungal Colonization of Wheat Grains with Fusarium spp. on Food Choice, Weight Gain and Mortality of Meal Beetle Larvae (Tenebrio molitor). PLoS ONE.

[148] Van Broekhoven S., Gutierrez J.M., De Rijk T., De Nijs W., Van Loon J. (2017). Degradation and excretion of the Fusarium toxin deoxynivalenol by an edible insect, the Yellow mealworm (Tenebrio molitor L.). World Mycotoxin J..

[149] De Zutter N., Audenaert K., Arroyo-Manzanares N., De Boevre M., Van Poucke C., De Saeger S., Haesaert G., Smagghe G. (2016). Aphids transform and detoxify the mycotoxin deoxynivalenol via a type II biotransformation mechanism yet unknown in animals. Sci. Rep..

[150] Camenzuli L., Van Dam R., De Rijk T., Andriessen R., Van Schelt J., Van Der Fels-Klerx H.J. (2018). (Ine) Tolerance and Excretion of the Mycotoxins Aflatoxin B1, Zearalenone, Deoxynivalenol, and Ochratoxin A by Alphitobius diaperinus and Hermetia illucens from Contaminated Substrates. Toxins.

[151] Berenbaum M.R., Bush D.S., Liao L.-H. (2021). Cytochrome P450-mediated mycotoxin metabolism by plant-feeding insects. Curr. Opin. Insect Sci..

[152] Stergiopoulos I., Gordon T.R. (2014). Cryptic fungal infections: The hidden agenda of plant pathogens. Front. Plant Sci..

[153] Marsberg A., Kemler M., Jami F., Nagel J.H., Postma-Smidt A., Naidoo S., Wingfield M.J., Crous P.W., Spatafora J.W., Hesse C.N. (2017). Botryosphaeria dothidea: A latent pathogen of global importance to woody plant health. Mol. Plant Pathol..

[154] Vega F.E. (2018). The use of fungal entomopathogens as endophytes in biological control: A review. Mycologia.

[155] Zhang Y., Han T., Ming Q., Wu L., Rahman K., Qin L. (2012). Alkaloids Produced by Endophytic Fungi: A Review. Nat. Prod. Commun..

[156] Schulz B., Haas S., Junker C., Andrée N., Schobert M. (2015). Fungal endophytes are involved in multiple balanced antago-nisms. Curr. Sci..

[157] Gange A.C., Koricheva J., Currie A.F., Jaber L.R., Vidal S. (2019). Meta-analysis of the role of entomopathogenic and unspecialized fungal endophytes as plant bodyguards. New Phytol..

[158] Bush L.P., Wilkinson H.H., Schardl C.L. (1997). Bioprotective Alkaloids of Grass-Fungal Endophyte Symbioses. Plant Physiol..

[159] Potter D.A., Stokes J.T., Redmond C.T., Schardl C.L., Panaccione D.G. (2008). Contribution of ergot alkaloids to suppression of a grass-feeding caterpillar assessed with gene knockout endophytes in perennial ryegrass. Èntomol. Exp. Appl..

[160] Schardl C.L., Young C., Hesse U., Amyotte S.G., Andreeva K., Calie P.J., Fleetwood D.J., Haws D.C., Moore N., Oeser B. (2013). Plant-Symbiotic Fungi as Chemical Engineers: Multi-Genome Analysis of the Clavicipitaceae Reveals Dynamics of Alkaloid Loci. PLoS Genet..

[161] Wilkinson H.H., Siegel M.R., Blankenship J.D., Mallory A.C., Bush L.P., Schardl C.L. (2000). Contribution of Fungal Loline Alkaloids to Protection from Aphids in a Grass-Endophyte Mutualism. Mol. Plant-Microbe Interact..

[162] Popay A., Tapper B., Podmore C. (2009). Endophyte infected meadow fescue and loline alkaloids affect Argentine stem weevil larvae. N. Z. Plant Prot..

[163] Koulman A., Lane G.A., Christensen M.J., Fraser K., Tapper B.A. (2007). Peramine and other fungal alkaloids are exuded in the guttation fluid of endophyte-infected grasses. Phytochemistry.

[164] Blankenship J.D., Spiering M., Wilkinson H.H., Fannin F.F., Bush L.P., Schardl C.L. (2001). Production of loline alkaloids by the grass endophyte, Neotyphodium uncinatum, in defined media. Phytochemistry.

[165] Watt G.A., Fleming R.A., Smith S.M., Fortin M.-J. (2018). Spruce Budworm (Choristoneura fumiferana Clem.) Defoliation Promotes Vertical Fuel Continuity in Ontario’s Boreal Mixedwood Forest. Forests.

[166] Miller J.D., MacKenzie S., Foto M., Adams G.W., Findlay J.A. (2002). Needles of white spruce inoculated with rugulosin-producing endophytes contain rugulosin reducing spruce budworm growth rate. Mycol. Res..

[167] Sumarah M.W., Miller J.D. (2009). Anti-Insect Secondary Metabolites from Fungal Endophytes of Conifer Trees. Nat. Prod. Commun..

[168] Stierle A.A., Stierle D.B. (2015). Bioactive Secondary Metabolites Produced by the Fungal Endophytes of Conifers. Nat. Prod. Commun..

[169] Findlay J.A., Buthelezi S., Lavoie R., Peña-Rodriguez L., Miller J.D. (1995). Bioactive Isocoumarins and Related Metabolites from Conifer Endophytes. J. Nat. Prod..

[170] Calhoun L.A., Findlay J.A., Miller J.D., Whitney N.J. (1992). Metabolites toxic to spruce budworm from balsam fir needle endophytes. Mycol. Res..

[171] Claydon N., Grove J.F., Pople M. (1985). Elm bark beetle boring and feeding deterrents from Phomopsis oblonga. Phytochemistry.

[172] Tanney J.B., McMullin D., Green B.D., Miller J.D., Seifert K.A. (2016). Production of antifungal and antiinsectan metabolites by the Picea endophyte Diaporthe maritima sp. nov. Fungal Biol..

[173] Thakur A., Singh V., Kaur A., Kaur S. (2013). Insecticidal potential of an endophytic fungus, Cladosporium uredinicola, against Spodoptera litura. Phytoparasit..

[174] Ondeyka J.G., Helms G.L., Hensens O.D., Goetz M.A., Zink D.L., Tsipouras A., Shoop W.L., Slayton L., Dombrowski A.W., Polishook J.D. (1997). Nodulisporic Acid A, a Novel and Potent Insecticide from a Nodulisporium sp. Isolation, Structure Determination, and Chemical Transformations. J. Am. Chem. Soc..

[175] Vinale F., Nicoletti R., Lacatena F., Marra R., Sacco A., Lombardi N., D’Errico G., Digilio M.C., Lorito M., Woo S.L. (2017). Secondary metabolites from the endophytic fungus Talaromyces pinophilus. Nat. Prod. Res..

[176] Ratnaweera P.B., Jayasundara J.M.N.M., Herath H.H.M.S.D., Williams D.E., Rajapaksha S.U., Nishantha K.M.D.W.P., de Silva E.D., Andersen R.J. (2020). Antifeedant, contact toxicity and oviposition deterrent effects of phyllostine acetate and phyllostine isolated from the endophytic fungus Diaporthe miriciae against Plutella xylostella larvae. Pest Manag. Sci..

[177] Berestetskiy A.O., Grigoryeva E.N., Petrova M.O., Stepanycheva E.A. (2018). Insecticidal and phytotoxic activity of extracts from cultures of some cereal pathogens. Mikologiya i Fitopatologiya.

[178] Robeson D.J., Strobel G.A. (1982). Monocerin, a Phytotoxin from Exserohilum turcicum (≡Drechslera turcica). Agric. Biol. Chem..

[179] Khambhati V.H., Abbas H.K., Sulyok M., Tomaso-Peterson M., Shier W.T. (2020). First Report of the Production of Mycotoxins and Other Secondary Metabolites by Macrophomina phaseolina (Tassi) Goid. Isolates from Soybeans (*Glycine max* L.) Symptomatic with Charcoal Rot Disease. J. Fungi.

[180] Gao W.-B., Liu S.-S., Tian Y.-C., Dong Q., Zhang J., Qin J.-C., Luo D.-Q. (2019). Setosphaerine A: A New Chlorinated Polyketide Isolated From the Entomogenous Fungus *Setosphaeria rostrata*. Nat. Prod. Comm..

[181] Bachmann T.L., Heide M., Manker D.C., Nielsen R.I., Rosendahl C.N. (1998). Fungicidal and insecticidal compounds and compositions derived from fungal strains of Pyrenophora teres. European Patent.

[182] Wicklow D.T., Rogers K.D., Dowd P.F., Gloer J.B. (2011). Bioactive metabolites from Stenocarpella maydis, a stalk and ear rot pathogen of maize. Fungal Biol..

[183] Aznar-Fernández T., Cimmino A., Masi M., Rubiales D., Evidente A. (2018). Antifeedant activity of long-chain alcohols, and fungal and plant metabolites against pea aphid (Acyrthosiphon pisum) as potential biocontrol strategy. Nat. Prod. Res..

[184] Masi M., Cimmino A., Tabanca N., Becnel J.J., Bloomquist J.R., Evidente A. (2017). A survey of bacterial, fungal and plant metabolites against Aedes aegypti (Diptera: Culicidae), the vector of yellow and dengue fevers and Zika virus. Open Chem..

[185] Cimmino A., Andolfi A., Avolio F., Ali A., Tabanca N., Khan I.A., Evidente A. (2013). Cyclopaldic Acid, Seiridin, and Sphaeropsidin A as Fungal Phytotoxins, and Larvicidal and Biting Deterrents against Aedes aegypti (Diptera: Culicidae): Structure-Activity Relationships. Chem. Biodivers..

[186] Cimmino A., Evidente M., Masi M., Ali A., Tabanca N., Khan I.A., Evidente A. (2015). Papyracillic acid and its derivatives as biting deterrents against Aedes aegypti (Diptera: Culicidae): Structure–activity relationships. Med. Chem. Res..

[187] Christias C., Hatzipapas P., Dara A., Kaliafas A., Chrysanthis G. (2001). Alternaria alternata, a new pathotype pathogenic to aphids. BioControl.

[188] Sharma I., Sharma A. (2014). Use of Alternaria spp. as a pest control agent: A review. World Appl. Sci. J..

[189] Rostás M., Hilker M. (2002). Asymmetric plant-mediated cross-effects between a herbivorous insect and a phytopathogenic fungus. Agric. For. Èntomol..

[190] Berestetskiy A.O., Apollonova L.S., Sokornova S.V., Chermenskaya T.D. (2015). Insecticidal properties of phytopathogenic ascomycetes. Vestnik Zashchity Rastenij.

[191] Singh B., Thakur A., Kaur S., Chadha B.S., Kaur A. (2012). Acetylcholinesterase Inhibitory Potential and Insecticidal Activity of an Endophytic Alternaria sp. from Ricinus communis. Appl. Biochem. Biotechnol..

[192] Kaur H.P., Singh B., Thakur A., Kaur A., Kaur S. (2015). Studies on immunomodulatory effect of endophytic fungus Alternaria alternata on Spodoptera litura. J. Asia-Pac. Èntomol..

[193] Kaur J., Sharma A., Sharma M., Manhas R.K., Kaur S., Kaur A. (2019). Effect of α-glycosidase inhibitors from endophytic fungus Alternaria destruens on survival and development of insect pest Spodoptera litura Fab. and fungal phytopathogens. Sci. Rep..

[194] Berestetskiy A.O., Gannibal F.B., Minkovich E.V., Osterman I.A., Salimova D.R., Sergiev P.V., Sokornova S.V. (2018). Spectrum of biological activity of the *Alternaria* fungi isolated from the phyllosphere of herbaceous plants. Mikrobiologiya.

[195] Buchwaldt L., Green H. (1992). Phytotoxicity of destruxin B and its possible role in the pathogenesis of Alternaria brassicae. Plant Pathol..

[196] Sree K.S., Padmaja V., Murthy Y.L.N. (2008). Insecticidal activity of destruxin, a mycotoxin fromMetarhizium anisopliae (Hypocreales), againstSpodoptera litura (Lepidoptera: Noctuidae) larval stages. Pest Manag. Sci..

[197] La Croix E.A.S., Mhasalkar S.E., Mamalis P., Harrington F.P. (1975). Insecticidal activity of some tenuazonic acid analogues. Pestic. Sci..

[198] Yang F.-Z., Yang B., Li B.-B., Xiao C. (2015). Alternaria toxin-induced resistance in rose plants against rose aphid (Macrosiphum rosivorum): Effect of tenuazonic acid. J. Zhejiang Univ. Sci. B.

[199] Yang F.-Z., Li Y.-X., Tang M., Zhu G.-L., Zhou S.-P., Yang B. (2020). Tenuazonic acid-induced change in volatile emission from rose plants and its chemometrical analysis. J. Plant Dis. Prot..

[200] Dalinova A., Chisty L., Kochura D., Garnyuk V., Petrova M., Prokofieva D., Yurchenko A., Dubovik V., Ivanov A., Smirnov A. (2020). Isolation and Bioactivity of Secondary Metabolites from Solid Culture of the Fungus, Alternaria sonchi. Biomolecules.

[201] Boddy L., Jones T.H. (2008). Chapter 9 Interactions between basidiomycota and invertebrates. Br. Mycol. Soc. Symp. Ser..

[202] Wieland T. (1996). Toxins and Psychoactive Compounds from Mushrooms. Human and Animal Relationships.

[203] Spiteller P. (2008). Chemical Defence Strategies of Higher Fungi. Chem. A Eur. J..

[204] Barseghyan G.S., Barazani A., Wasser S.P. (2016). Medicinal Mushrooms with Anti-Phytopathogenic and Insecticidal Properties. Mushroom Biotechnol..

[205] Mier N., Canete S., Klaebe A., Chavant L., Fournier D. (1996). Insecticidal properties of mushroom and toadstool carpophores. Phytochemistry.

[206] Sargazi F., Riahi H., Sheidai M. (2009). Insecticidal Activities of Ling Zhi or Reishi Medicinal Mushroom, Ganoderma lucidum (W. Curt.: Fr.) P. Karst. (Aphyllophoromycetideae) Extract against Tribolium castaneum and Drosophila melanogaster. Int. J. Med. Mushrooms.

[207] Schachter E., Zuskin E., Goswami S., Castranova V., Arumugam U., Whitmer M., Siegel P., Chiarelli A., Fainberg J. (2005). Pharmacological Study of Oyster Mushroom (Pleurotus Ostreatus) Extract on Isolated Guinea Pig Trachea Smooth Muscle. Lung.

[208] Masota N.E., Sempombe J., Mihale M., Henry L., Mugoyela V., Sung’hwa F. (2017). Pesticidal activity of wild mushroom Cantharellus cibarius (FR) extracts against Sitophilus zeamais (Motschulsky) (Coleoptera: Curculionidae) in stored maize grains. J. Food Secur..

[209] Sivanandhan S., Khusro A., Paulraj M.G., Ignacimuthu S., Al-Dhabi N.A. (2017). Biocontrol Properties of Basidiomycetes: An Overview. J. Fungi.

[210] Njogu E., Njue A., Omolo J., Cheplogoi P. (2010). Larvicidal activity of (oxiran-2-yl)methylpentanoate extracted from mushroom Cyptotrama asprata against mosquito Aedes aegypti. Int. J. Biol. Chem. Sci..

[211] Stadler M., Sterner O. (1998). Production of bioactive secondary metabolites in the fruit bodies of macrofungi as a response to injury. Phytochem..

[212] Brandt P., García-Altares M., Nett M., Hertweck C., Hoffmeister D. (2017). Induced Chemical Defense of a Mushroom by a Double-Bond-Shifting Polyene Synthase. Angew. Chem. Int. Ed..

[213] Gagkaeva T.Y., Shamshev I.V., Gavrilova O.P., Selitskaya O.G. (2014). Biological Relationships Between Fusarium Fungi and Insects (Review). Sel’skokhozyaistvennaya Biologiya [Agric. Biol.].

[214] Connick W.J., French R.C. (1991). Volatiles emitted during the sexual stage of the Canada thistle rust fungus and by thistle flowers. J. Agric. Food Chem..

[215] Fäldt J., Jonsell M., Nordlander G., Borg-Karlson A.-K. (1999). Volatiles of Bracket Fungi Fomitopsis pinicola and Fomes fomentarius and Their Functions as Insect Attractants. J. Chem. Ecol..

[216] Hung R., Lee S., Bennett J.W. (2015). Fungal volatile organic compounds and their role in ecosystems. Appl. Microbiol. Biotechnol..

[217] Beck J.J., Vannette R. (2017). Harnessing Insect–Microbe Chemical Communications To Control Insect Pests of Agricultural Systems. J. Agric. Food Chem..

[218] Mitina G.V., Selitskaya O.G., Schenikova A.V. (2020). Effect of Volatile Compounds of the Entomopathogenic Fungi Beauveria bassiana (Bals.-Criv.) Vuill. and Lecanicillium muscarium R. Zare et W. Gams on the Behavior of Sitophilus granarius (L.) (Coleoptera, Dryophthoridae) and Evaluation of the Virulence of Different Strains of These Fungi. Èntomol. Rev..

[219] Lozano-Soria A., Picciotti U., Lopez-Moya F., Lopez-Cepero J., Porcelli F., Lopez-Llorca L.V. (2020). Volatile Organic Compounds from Entomopathogenic and Nematophagous Fungi, Repel Banana Black Weevil (*Cosmopolites sordidus*). Insects.

[220] Bojke A., Tkaczuk C., Stepnowski P., Gołębiowski M. (2018). Comparison of volatile compounds released by entomopathogenic fungi. Microbiol. Res..

[221] Daisy B.H., Strobel G.A., Castillo U., Ezra D., Sears J., Weaver D.K., Runyon J.B. (2002). Naphthalene, an insect repellent, is produced by Muscodor vitigenus, a novel endophytic fungus. Microbiology.

[222] Nakajima H., Ishihara A., Sawa Y., Sakuno E. (2010). 3-(4-Methylfuran-3-yl)propan-1-ol: A White-Spotted Stinkbug (Eysarcoris ventralis) Repellent Produced by an Endophyte Isolated from Green Foxtail. J. Agric. Food Chem..

[223] Quesada-Moraga E., Carrasco-Diaz J.-A., Santiago-Alvarez C. (2006). Insecticidal and antifeedant activities of proteins secreted by entomopathogenic fungi against Spodoptera littoralis (Lep., Noctuidae). J. Appl. Èntomol..

[224] Quesada-Moraga E., Vey A. (2004). Bassiacridin, a protein toxic for locusts secreted by the entomopathogenic fungus Beauveria bassiana. Mycol. Res..

[225] Khan S., Nadir S., Lihua G., Xu J., Holmes K.A., Dewen Q. (2016). Identification and characterization of an insect toxin protein, Bb70p, from the entomopathogenic fungus, Beauveria bassiana, using Galleria mellonella as a model system. J. Invertebr. Pathol..

[226] Ortiz-Urquiza A., Garrido-Jurado I., Santiago-Álvarez C., Quesada-Moraga E. (2009). Purification and characterization of proteins secreted by the entomopathogenic fungus Metarhizium anisopliae with insecticidal activity against adults of the Mediterranean fruit fly, Ceratitis capitata (Diptera: Tephritidae). Pest Manag. Sci..

[227] Ortiz-Urquiza A., Riveiro-Miranda L., Santiago-Álvarez C., Quesada-Moraga E. (2010). Insect-toxic secreted proteins and virulence of the entomopathogenic fungus Beauveria bassiana. J. Invertebr. Pathol..

[228] Ortiz-Urquiza A., Keyhani N.O., Quesada-Moraga E. (2013). Culture conditions affect virulence and production of insect toxic proteins in the entomopathogenic fungus Metarhizium anisopliae. Biocontrol Sci. Technol..

[229] Herrero-Galán E., Lacadena J., del Pozo Á.M., Boucias D.G., Olmo N., Oñaderra M., Gavilanes J.G. (2008). The insecticidal protein hirsutellin A from the mite fungal pathogen Hirsutella thompsoniiis a ribotoxin. Proteins Struct. Funct. Bioinform..

[230] Li H., Xia Y. (2018). High cell density fed-batch production of insecticidal recombinant ribotoxin hirsutellin A from Pichia pastoris. Microb. Cell Factories.

[231] Olombrada M., Medina P., Budia F., Gavilanes J.G., Martínez-Del-Pozo Á., García-Ortega L. (2017). Characterization of a new toxin from the entomopathogenic fungus Metarhizium anisopliae: The ribotoxin anisoplin. Biol. Chem..

[232] Hamshou M., Van Damme E., Smagghe G. (2010). Entomotoxic effects of fungal lectin from Rhizoctonia solani towards Spodoptera littoralis. Fungal Biol..

[233] Hamshou M., Smagghe G., Shahidi-Noghabi S., De Geyter E., Lannoo N., Van Damme E.J. (2010). Insecticidal properties of Sclerotinia sclerotiorum agglutinin and its interaction with insect tissues and cells. Insect Biochem. Mol. Biol..

[234] Wang M., Triguéros V., Paquereau L., Chavant L., Fournier D. (2002). Proteins as Active Compounds Involved in Insecticidal Activity of Mushroom Fruitbodies. J. Econ. Èntomol..

[235] Avanzo P., Sabotič J., Anžlovar S., Popovič T., Leonardi A., Pain R.H., Kos J., Brzin J. (2009). Trypsin-specific inhibitors from the basidiomycete Clitocybe nebularis with regulatory and defensive functions. Microbiology.

[236] Pohleven J., Brzin J., Vrabec L., Leonardi A., Čokl A., Štrukelj B., Kos J., Sabotič J. (2011). Basidiomycete Clitocybe nebularis is rich in lectins with insecticidal activities. Appl. Microbiol. Biotechnol..

[237] Citores L., Ragucci S., Ferreras J.M., Di Maro A., Iglesias R. (2019). Ageritin, a Ribotoxin from Poplar Mushroom (Agrocybe aegerita) with Defensive and Antiproliferative Activities. ACS Chem. Biol..

[238] Tayyrov A., Azevedo S., Herzog R., Vogt E., Arzt S., Lüthy P., Müller P., Rühl M., Hennicke F., Künzler M. (2019). Heterologous Production and Functional Characterization of Ageritin, a Novel Type of Ribotoxin Highly Expressed during Fruiting of the Edible Mushroom Agrocybe aegerita. Appl. Environ. Microbiol..

[239] Baglivo I., Ragucci S., D’Incecco P., Landi N., Russo R., Faoro F., Pedone P., Di Maro A. (2020). Gene Organization, Expression, and Localization of Ribotoxin-Like Protein Ageritin in Fruiting Body and Mycelium of *Agrocybe aegerita*. Int. J. Mol. Sci..

[240] Langenfeld A., Blond A., Gueye S., Herson P., Nay B., Dupont J., Prado S. (2011). Insecticidal Cyclodepsipeptides from Beauveria felina. J. Nat. Prod..

[241] Chen D., Zhang P., Liu T., Wang X.-F., Li Z.-X., Li W., Wang F.-L. (2018). Insecticidal Activities of Chloramphenicol Derivatives Isolated from a Marine Alga-Derived Endophytic Fungus, Acremonium vitellinum, against the Cotton Bollworm, Helicoverpa armigera (Hübner) (Lepidoptera: Noctuidae). Molecules.

[242] Bai M., Zheng C.-J., Nong X.-H., Zhou X.-M., Luo Y.-P., Chen G.-Y. (2019). Four New Insecticidal Xanthene Derivatives from the Mangrove-Derived Fungus Penicillium sp. JY246. Mar. Drugs.

[243] Zhang P., Yuan X.-L., Du Y.-M., Zhang H.-B., Shen G.-M., Zhang Z.-F., Liang Y.-J., Zhao D.-L., Xu K. (2019). Angularly Prenylated Indole Alkaloids with Antimicrobial and Insecticidal Activities from an Endophytic Fungus Fusarium sambucinum TE-6L. J. Agric. Food Chem..

[244] Mao Z., Wang W., Su R., Gu G., Liu Z.L., Lai D., Zhou L. (2019). Hyalodendrins A and B, New Decalin-Type Tetramic Acid Larvicides from the Endophytic Fungus Hyalodendriella sp. Ponipodef12. Molecules.

[245] Yuan X.-L., Wang X.-F., Xu K., Li W., Chen D., Zhang P. (2020). Characterization of a New Insecticidal Anthraquinone Derivative from an Endophyte of Acremonium vitellinum against Helicoverpa armigera. J. Agric. Food Chem..

[246] Berestetskiy A.O., Dalinova A.A., Dubovik V.R., Grigoryeva E.N., Kochura D.M., Senderskiy I.V., Smirnov S.N., Stepanycheva E.A., Turaeva S.M. (2020). Analysis and Isolation of Secondary Metabolites of Bipolaris sorokiniana by Different Chromatography Techniques and the Spectrum of Their Biological Activity. Appl. Biochem. Microbiol..

[247] Dalinova A., Dubovik V., Chisty L.S., Kochura D.M., Ivanov A.Y., Smirnov A., Petrova M.O., Zolotarev A.A., Evidente A., Berestetskiy A. (2019). Stagonolides J and K and Stagochromene A, Two New Natural Substituted Nonenolides and a New Disubstituted Chromene-4,5-dione Isolated from Stagonospora cirsii S-47 Proposed for the Biocontrol of Sonchus arvensis. J. Agric. Food Chem..

[248] Li B., Yuan H., Fang J., Tao L., Huang Q., Qian X., Fan Z. (2009). Recent progress of highly efficient in vivo biological screening for novel agrochemicals in China. Pest Manag. Sci..

[249] Hyun S.-H. (2013). Alteration of Media Composition and Light Conditions Change Morphology, Metabolic Profile, and Beauvericin Biosynthesis in Cordyceps bassiana Mycelium. J. Microbiol. Biotechnol..

[250] Hyun S.-H., Lee S.-Y., Sung G.-H., Kim S.H., Choi H.-K. (2013). Metabolic Profiles and Free Radical Scavenging Activity of Cordyceps bassiana Fruiting Bodies According to Developmental Stage. PLoS ONE.

[251] Matsuda D., Namatame I., Tomoda H., Kobayashi S., Zocher R., Kleinkauf H., Omura S. (2004). Beauveriolides Produced by Amino Acid-supplemented Fermentation of Beauveria sp. FO-6979. J. Antibiot..

[252] De Bekker C., Smith P.B., Patterson A., Hughes D.P. (2013). Metabolomics Reveals the Heterogeneous Secretome of Two Entomopathogenic Fungi to Ex Vivo Cultured Insect Tissues. PLoS ONE.

[253] Yin H.-Y., Yang X.-Q., Wang D.-L., Zhao T.-D., Wang C.-F., Yang Y.-B., Ding Z.-T. (2021). Antifeedant and antiphytopathogenic metabolites from co-culture of endophyte Irpex lacteus, phytopathogen Nigrospora oryzae, and entomopathogen Beauveria bassiana. Fitoterapia.

[254] Arroyo-Manzanares N., Di Mavungu J.D., Garrido-Jurado I., Arce L., Vanhaecke L., Quesada-Moraga E., De Saeger S. (2017). Analytical strategy for determination of known and unknown destruxins using hybrid quadrupole-Orbitrap high-resolution mass spectrometry. Anal. Bioanal. Chem..

[255] Xiao G., Ying S.-H., Zheng P., Wang Z.-L., Zhang S., Xie X.-Q., Shang Y., Leger R.J.S., Zhao G.-P., Wang C. (2012). Genomic perspectives on the evolution of fungal entomopathogenicity in Beauveria bassiana. Sci. Rep..

[256] Gibson D.M., Donzelli B., Krasnoff S.B., Keyhani N.O. (2014). Discovering the secondary metabolite potential encoded within entomopathogenic fungi. Nat. Prod. Rep..

[257] Bignell E., Cairns T., Throckmorton K., Nierman W.C., Keller N.P. (2016). Secondary metabolite arsenal of an opportunistic pathogenic fungus. Philos. Trans. R. Soc. B Biol. Sci..

[258] Tsukada K., Shinki S., Kaneko A., Murakami K., Irie K., Murai M., Miyoshi H., Dan S., Kawaji K., Hayashi H. (2020). Synthetic biology based construction of biological activity-related library of fungal decalin-containing diterpenoid pyrones. Nat. Commun..

[259] Sansinenea E., Ortiz A. (2011). Secondary metabolites of soil *Bacillus* spp.. Biotechnol. Lett..

[260] Ortiz A. (2013). An Antibiotic from Bacillus thuringiensis against Gram-Negative Bacteria. Biochem. Pharmacol..

[261] Liu X., Ruan L., Peng D., Li L., Sun M., Yu Z. (2014). Thuringiensin: A Thermostable Secondary Metabolite from Bacillus thuringiensis with Insecticidal Activity against a Wide Range of Insects. Toxins.

[262] Zhao H., Lovett B., Fang W. (2016). Genetically Engineering Entomopathogenic Fungi. Fungal Genom..

[263] Leger R.S., Joshi L., Bidochka M.J., Roberts D.W. (1996). Construction of an improved mycoinsecticide overexpressing a toxic protease. Proc. Natl. Acad. Sci. USA.

[264] Wang C., Leger R.J.S. (2007). A scorpion neurotoxin increases the potency of a fungal insecticide. Nat. Biotechnol..

[265] Xie M., Zhang Y.-J., Zhai X.-M., Zhao J.-J., Peng D.-L., Wu G. (2015). Expression of a scorpion toxin gene BmKit enhances the virulence of Lecanicillium lecanii against aphids. J. Pest Sci..

[266] Deng S.-Q., Cai Q.-D., Deng M.-Z., Huang Q., Peng H.-J. (2017). Scorpion neurotoxin AaIT-expressing Beauveria bassiana enhances the virulence against Aedes albopictus mosquitoes. AMB Express.

[267] Qin Y., Ying S.-H., Chen Y., Shen Z.-C., Feng M.-G. (2010). Integration of Insecticidal Protein Vip3Aa1 into Beauveria bassiana Enhances Fungal Virulence to Spodoptera litura Larvae by Cuticle and Per Os Infection. Appl. Environ. Microbiol..

[268] Deng S.-Q., Zou W.-H., Li D.-L., Chen J.-T., Huang Q., Zhou L.-J., Tian X.-X., Chen Y.-J., Peng H.-J. (2019). Expression of Bacillus thuringiensis toxin Cyt2Ba in the entomopathogenic fungus Beauveria bassiana increases its virulence towards Aedes mosquitoes. PLoS Negl. Trop. Dis..

